# Meta-analysis of the impact of future climate change on the area of woody plant habitats in China

**DOI:** 10.3389/fpls.2023.1139739

**Published:** 2023-03-15

**Authors:** Pingping Tian, Yifu Liu, Jing Ou

**Affiliations:** ^1^ College of Forestry, Guizhou University, Guiyang, China; ^2^ Key Laboratory of Forest Ecology and Environment of National Forestry and Grassland Administration, Ecology and Nature Conservation Institute, Chinese Academy of Forestry, Beijing, China

**Keywords:** climate change, meta-analysis, woody plant, habitat area, China

## Abstract

Climate change poses a very serious threat to woody plants, and it is important to study its impact on the distribution dynamics of woody plants in China. However, there are no comprehensive quantitative studies on which factors influence the changes in the area of woody plant habitats in China under climate change. In this meta-analysis, we investigated the future suitable habitat area changes of 114 woody plant species in 85 studies based on MaxEnt model predictions to summarize the future climate change impacts on woody plant habitat area changes in China. It was found that climate change will result in a 3.66% increase in the overall woody plant suitable areas and a 31.33% decrease in the highly suitable areas in China. The mean temperature of the coldest quarter is the most important climatic factor, and greenhouse gas concentrations were inversely related to the area of future woody plant suitable areas. Meanwhile, shrubs are more climate-responsive than trees, drought-tolerant plants (e.g., *Dalbergia*, *Cupressus*, and *Xanthoceras*) and plants that can adapt quickly (e.g., *Camellia, Cassia*, and *Fokienia*) and their appearance will increase in the future. Old World temperate, Trop. Asia and Trop. Amer. disjuncted, and the Sino-Himalaya Floristic region are more vulnerable. Quantitative analysis of the possible risks to future climate change in areas suitable for woody plants in China is important for global woody plant diversity conservation.

## 1 Introduction

Over the past century, the planet has warmed by 1.1°C, and this warming is projected to exceed 1.5°C in the coming decades even with very low greenhouse gas (GHG) emissions (Rutledge, 2011; Pörtner et al., 2022). Current climate change has already led to a significant reduction in forest vegetation globally, with the continued movement of species to polar and high-altitude regions (McKenney et al., 2007; Leadley et al., 2010; Zhou et al., 2014; Greenwood et al., 2017; Pecl et al., 2017; Anchang et al., 2019). Such impacts will continue to increase under future climate change and will outweigh human impacts causing habitat destruction (Hewitt, 2000; Parmesan et al., 2000; Parmesan and Yohe, 2003; Engler et al., 2011; Pecl et al., 2017). China is one of the most biodiversity-rich countries in the world, but under the disturbance of climate change, China has also become one of the countries with the most threatened biodiversity in the world (Mcneely et al., 1990; Su et al., 2018; Peng et al., 2022). Among the more than 30,000 species of higher plants in China, woody plants (defined as those that maintain prominent aboveground stems that persist over time and under changing environmental conditions) (Zanne et al., 2014) account for almost one-third of its total plants (Tang et al., 2006; Xie et al., 2021). Climate change poses a very serious threat to woody plants, with extinction rates reaching 30% in extreme cases (Zhang et al., 2014; Ahmed et al., 2015; Koo et al., 2015). Bezeng et al. (2017) simulated the potential future ranges of 162 species of woody plants in South Africa and found that more than 50% of the suitable areas for these species will be reduced. Therefore, understanding the future dynamics of woody plants in China is of great importance for global biodiversity conservation.

Species distributions and habitat areas represent the constraints and dispersal effects of environmental factors (Brown et al., 1996). The effects of factors such as temperature, CO_2_ concentration and climate on the growth of woody plants have been the subject of several studies (Zanne et al., 2014; Baig et al., 2015; Zhang et al., 2016; Liu et al., 2018; Peng et al., 2021). To further investigate the effects of climate change on plant distribution dynamics, several research have used species distribution models (SDMs) to simulate changes in the geographic distributions and areas of plants (Guisan and Thuiller, 2005; Alo and Wang, 2008; Valavi et al., 2022) to study the effects on species distribution patterns, which is essential for developing strategies for future biodiversity conservation (Bellard et al., 2012). In addition, to further achieve China’s goal of ‘peak carbon and carbon neutrality’, a series of emission reduction measures have been implemented, with forest carbon sequestration being one of the key directions (Wei et al., 2022). The species distribution model predicts suitable distribution patterns for regional tree species, based on which species biomass, carbon stocks and carbon sequestration potential can be more effectively assessed (Wang et al., 2022). The MaxEnt model is widely used as the SDM with the best accuracy and software utility (Yan et al., 2021; Zhao et al., 2021; Wu et al., 2022), and as of 2019, 80.1% of 33 simulated plant distribution models have utilized the MaxEnt model (Merow et al., 2013; Ahmed et al., 2015; Liu et al., 2019). However, there are no comprehensive quantitative studies on which factors influence changes in the area of woody plant habitats in China under climate change and to what extent. Therefore, based on the MaxEnt model, this study synthesized 85 articles with 115 woody plant species and 959 effect sizes (528 effect sizes for the overall suitable areas and 431 effect sizes for the highly suitable areas) for meta-analysis to investigate the distribution of suitable areas for woody plants in China under current and future climate change. To address the important question of future climate change impacts on woody plants in China, we developed four questions and hypotheses. (a) Will future climate change negatively affect both overall and highly suitable areas? (b) What are the main climatic factors affecting future changes in the area of suitable areas? (c) Do higher concentrations of greenhouse gas emissions affect the size of the suitable zone? (d) Will woody plants of different attributes (life type, genus, and floristic region) be affected in approximately the same way. This study quantitatively analyses the future area of woody plant habitats in China to understand their trends under climate change and their main influencing factors and provide a basis for Chinese forest management authorities to develop effective measures to protect woody plants, regulate the adaptive capacity of natural systems and conserve biodiversity.

## 2 Materials and methods

### 2.1 Data sources

In this study, relevant data were collected through a systematic literature search for relevant studies published between 1990 and May 2022 through Web of Science, Google Scholar, and China National Knowledge Infrastructure (CNKI) on June 1, 2022, using the following keywords: “MaxEnt”, “climate change”, “future”, “ China”, “distribution”, and “plant”.

The selection criteria were as follows: (1) studies contain projections for woody plants within China and do not contain projections that were beyond the scope of China or smaller than the scope of China (e.g., local scope of China such as northwest, southeast, and Yunnan provinces of China); (2) studies contain models of only a single species individually using MaxEnt and not models of more than two species uniformly, such as a type or a family; (3) the studies use MaxEnt to model both contemporary and future climate change impacts on woody plant distribution dynamics and do not assess only contemporary or only future distribution dynamics; (4) the studies use area under the curve (AUC) values greater than 0.8 (high model accuracy); and (5) the studies document the contribution of climatic factors to changes in the distribution of woody plants.

**Figure 1:**
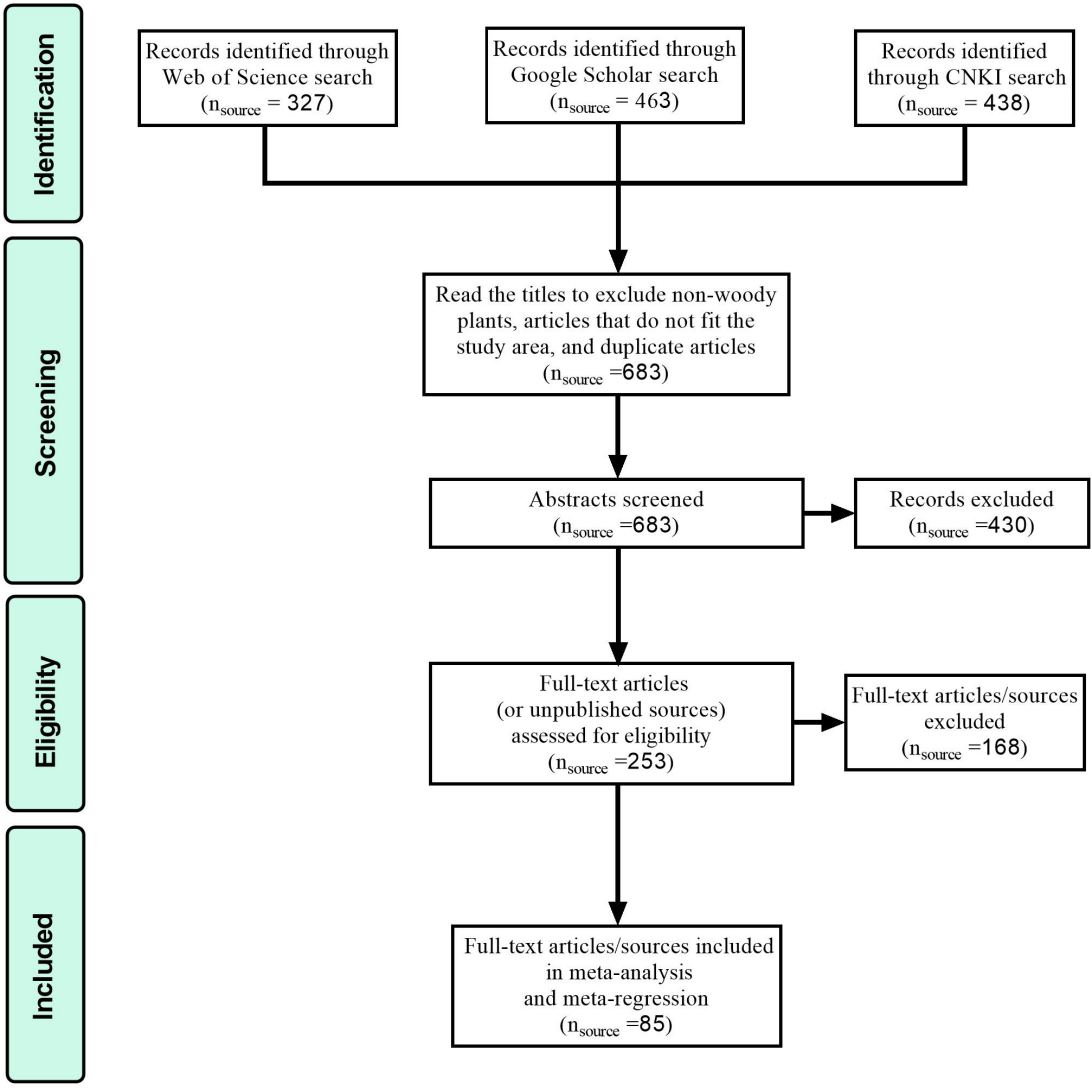
PRISMA flow chart showing the procedure for selecting publications.

We searched tens of thousands of articles by keywords, and we simply excluded studies that were not woody plants (animals, pests, herbs, etc.) by visually checking the study titles on the web pages and obtained 1228 studies for in-depth screening and inspection. A total of 1228 articles were searched through Web of Science, Google Scholar, and China National Knowledge Infrastructure (CNKI) to visually exclude articles on nonplant studies (e.g., animals, pests and diseases). First, since the word woody plants is not usually included directly in the articles, we read the titles to exclude articles with nonwoody plants, studies in areas larger or smaller than the scope of China (e.g., studies in northwestern China, southeastern China, Yunnan Province, and other local areas of China), and articles with duplicate contents and selected from these 1228 articles current and future studies on woody plants in China based on the MaxEnt model. From these 1228 articles, we selected 683 studies based on the MaxEnt model to simulate the current and future fitness zones of Chinese woody plants. Second, by reading the abstracts, we further excluded those studies that were not woody plants, those that studied areas larger or smaller than the Chinese range, and those that did not use the MaxEnt model or those that used the MaxEnt model but had an AUC value less than 0.8. A total of 253 studies of woody plants in China based on the MaxEnt model were screened out. Finally, by reading the full text, we excluded studies that used the same MaxEnt model to study multiple woody plants (e.g., the whole genus or a dozen plants combined), cultivated plants, studies that did not use climate factors for modelling, and studies that did not record the contribution of climate factors after modelling. Finally, 85 research papers meeting the criteria, including 64 studies in Chinese and 21 studies in English, were screened for meta-analysis (**Figure 1**).

For the literature that was selected, the following data were extracted: species names, sample sizes, GHG emission scenarios (representative concentration pathway (RCP) and shared socioeconomic pathway (SSP)) and emission concentrations of the studied plants (RCP scenarios included RCP2.6, RCP4.5, RCP6.0 and RCP8.5; SSP scenarios included SSP126, SSP245, SSP370, and SSP585). The numbers after RCP and SSP represent radiative forcings of approximately 8.5, 6.0, 4.5 and 2.6 W m^–2^ for the scenario, respectively, and the GHGs include CO_2_, N_2_O, CH_4_, CFC-11 and CFC-12, with the SSP adding socioeconomic development considerations compared to the RCP. (Zhang et al., 2013; Wang et al., 2019), the overall and highly suitable areas for the contemporary and future period predicted by MaxEnt modelling and the main climatic factors affecting the distribution of the plants and their contribution to their distribution (**Supplementary Table 1**). Data not directly expressed in the texts were extracted from charts in the papers using Web Plot Digitizer 4.5 (https://apps.automeris.io/wpd/index.zh_CN.html) and visually checked for accuracy after extraction. Data on the characteristics of the studied species were recorded by reviewing the data: plant life type (tree and shrub), plant genus (**Supplementary Table 2**), and floristic region. Since each study provided the mean, the standard deviation was 1/10 of the mean where no standard error, standard deviation, or confidence interval was reported (Luo et al., 2006). Finally, a total of 114 woody plant species belonging to 74 genera in 42 families and 19 floristic regions were collected and collated, and 959 sets of study data were extracted (overall suitable area: 114 species, 528 sets of effects; highly suitable region: 85 species, 431 sets of effects).

### 2.2 Research methods

The results of the literature were normalized by calculating effect sizes, which were expressed as log response ratios (Hedges et al., 1999; Lajeunesse, 2011):


(Eq. 1) $$y_{i}=\text{ln}\left(\frac{{\bar{x}}_{f}}{{\bar{x}}_{c}}\right)$$

In Equation 1, ${\bar{x}}_{f}$ is the average area of woody plants (km^2^) in China under future climate change based on MaxEnt modelling, and ${\bar{x}}_{c}$ is the average area of woody plants (km^2^) in the contemporary period based on MaxEnt modelling.

The formula for the within-case variance (*y_i_*) corresponding to the effect size (*v_i_*) was as follows:


(Eq. 2) $$v_{i}=\frac{S_{f}^{2}}{N_{f}Y_{f}}+\frac{S_{c}^{2}}{N_{c}Y_{c}}$$

In (Eq 2), (*v_i_*) denotes the within-case variance, (*S_f_*) denotes the standard deviation of ( ${\bar{x}}_{f}$ ), and (*S_c_*) denotes the standard deviation of ( ${\bar{x}}_{c}$ ), where (*N_f_*) and (*N_c_*) denote the sample size (Hedges et al., 1999; Lajeunesse, 2011).

The effect values obtained by meta-analysis and the percentage change in area were used as the results of the analysis, and the percentage change in area was calculated as follows:


$$m=\left(\exp\left(\ln\frac{{\bar{x}}_{f}}{{\bar{x}}_{C}}\right)-1\right)\times 100\%$$

where (*m*) is the percentage change in area.

### 2.3 Data analysis

To accurately assess the impact of future climate change on suitable woody plant areas, this study first assessed the overall response of the overall suitable area and highly suitable area separately using a random effects model and then introduced explanatory variables for a mixed effects meta-regression model to assess the impact of influencing factors (Su et al., 2021b). The overall suitable area and the highly suitable area are divided according to the suitability indices of different species in their respective MaxEnt model studies. The overall suitable area is the range that satisfies the basic survival conditions of plants, and the highly suitable area is the range where plants are in the best biophysiological condition. All analyses were performed in R 4.2.1 software, and all parameters were estimated using the restricted maximum likelihood method (REML)(Viechtbauer, 2010). The robustness of the Rosenberg fail-safe assessment results to publication bias was calculated using funnel plots and Egger regression tests for publication bias (Egger et al., 1997; Rosenberg, 2005).

## 3 Results and analysis

### 3.1 Model heterogeneity and reliability assessment

Compared to the control group, the overall suitable area increased by 3.66% (P< 0.05), but the highly suitable area decreased by 31.33% under climate change (P< 0.05). Heterogeneity was assessed by formal Cochran’s Q test for the overall suitable area dataset results (Qt = 286258.6337, df = 527, P< 0.0001) and the highly suitable region area dataset results (Qt = 20007718.4171, df = 430, P< 0.0001). Both the overall suitable area and the highly suitable area were greatly heterogeneous, so different influencing factors needed to be introduced to explain them. Therefore, this study incorporated the collected climatic factors, different concentrations of GHGs under the RCP and SSP scenarios, woody plant life types, woody plant genera, and the floristic region to which woody plants were fixed factors (moderators) for each response variable. These factors were then evaluated using a mixed-effects meta-regression model to explore the changes in response to these influences in the future suitable area for woody plants. If the 95% confidence interval (CI) did not overlap with zero and the significance was less than the 0.05 level, then the factor had a significant effect.

The results for the overall suitable region area were p = 0.3235 (>0.05), indicating that the results were less affected by publication bias and that the funnel plot shape was symmetrical (**Supplementary Figure 1**). The test results for the highly suitable area were p = 0.8865 (>0.05), indicating that the results were minimally affected by publication bias and that the funnel plot shape was symmetrical (**Supplementary Figure 2**). Rosenberg’s fail-safe numbers were for the overall suitable area (1062630, P< 0.0001) and the highly suitable region area (60754916, P< 0.0001), indicating reliable model results.

### 3.2 Trends in woody plant habitat areas in China under future climate change

The overall woody plant suitable area will significantly increase by 3.66% (95% confidence interval [CI]: 0.0089, 0.0629) under future climate change, with R^2^, Σ*R* ^2^ = 58.76% for all explanatory variables, indicating that the explanatory variables included in this study explain 58.76% of the sources of heterogeneity in the overall suitable area (**Supplementary Table 3**). The highly suitable area will be significantly reduced by 31.33% (95% confidence interval [CI]: −0.5967, −0.1548), and the R^2^, Σ*R* ^2^ = 42.78% for all explanatory variables, indicating that the explanatory variables included in this study explain 42.78% of the sources of heterogeneity in highly suitable areas (**Supplementary Table 3**). Therefore, future changes in woody plant area will be influenced by multiple factors, some of which were not included in the model (e.g., interspecific competition).

### 3.3 Response of the future woody plant habitat areas to different climatic factors in China

**Figure 2:**
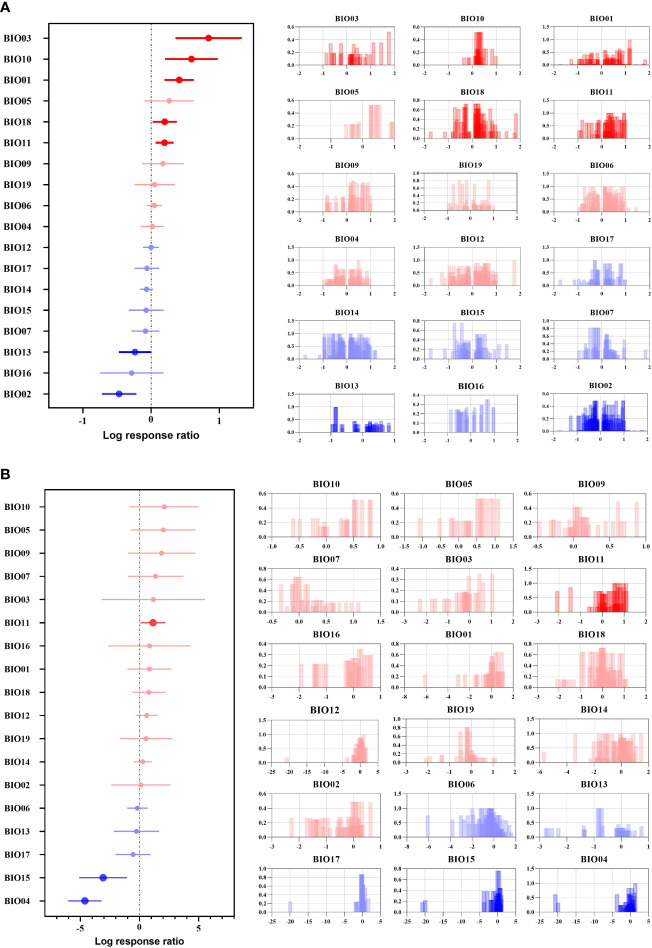
Effect sizes of different climate factors. **(A)** indicates the overall suitable area, and **(B)** indicates the highly suitable area. The left side represents the effect of 18 climate factors on the future overall/highly suitable area (main effects) for woody plants, and error lines indicate 95% confidence intervals. The right panel is a histogram of the impact of the contribution of each climate factor to the effect values. Positive effect (red color), significant positive effect (bright red color), negative effect (blue color), and significant negative effect (bright blue color).

For the overall suitable area, 11 climatic factors had a positive effect, and 5 climatic factors had a significant positive effect (**Figure 2A**). Isothermality (BIO03) had a significant overall positive effect on woody plants and an extreme positive effect on some woody plants, such as *Salix paraplegia* and *Dalbergia sericea* (corresponding to the contribution histogram in **Figure 2A**), while the strongest aggregation effect and the shortest error line occurred for precipitation of the warmest quarter (BIO18), mean temperature of the coldest quarter (BIO11) and mean temperature of the warmest quarter (BIO10), indicating that most Chinese woody plants will be affected by these three climate factors to the same extent and with the same trend in the future. The significant negative effect of the mean diurnal range (BIO02) indicates that the variation in BIO02 under future climate will be beyond the survival tolerance range of woody plants, such as *Sorbus amabilis*, *Cathaya argyrophylla* and *Ormosia hosiei*. Precipitation of the wettest month (BIO13) also had a significant negative effect, but most of the woody plant effects were clustered between 0 and 1, with individually significant variability from 0 to -1 (corresponding to the contribution histogram in **Figure 2A**), indicating that future changes in BIO13 will have a stronger negative effect than other factors on the fitness zone of some woody plants, such as Populus euphratica, *Rhododendron protistum* and *Osmanthus serrulatus.*

For the highly suitable area, 13 climate factors had positive effects, and 5 had negative effects (**Figure 2B**). Among them, BIO11 had a significant positive effect, indicating that BIO11 will reach the optimal survival temperature for Chinese woody plants under future climate change, suc*h a*s *Ziziphus spinosa*, *Lonicera japonica* and *Picea likiangensis*. While temperature seasonality (BIO04) and precipitation seasonality (BIO15) had significant negative effects, BIO04 and BIO15 were heavily clustered within the effect value of –5 (corresponding contribution histogram in **Figure 2B**), indicating that future changes in BIO04 and BIO15 will be beyond the tolerance range of most woody plants, resulting in a reduction in the highly suitable areas for woody plants in the future, such as *Ulmus pumila*, *Phoebe bournei* and *Ostrya rehderiana*. At the same time, BIO04 explained 8.59% of the variation in the effect values, and the explanation rate accounted for 70.00% of the explanation rate of all climatic factors influencing the area change of highly suitable areas in this study, indicating that BIO04 was the most important climatic factor influencing the future change in highly suitable areas for woody plants.

Overall, the climatic factors selected in this study explained 11.35% of the variation in the overall future woody plant suitable area and 12.13% of the variation in the highly suitable area, indicating that the highly suitable area for woody plants is more influenced by climatic factors.

### 3.4 Response of the future woody plant habitat areas to different GHG emission scenarios in China

**Figure 3:**
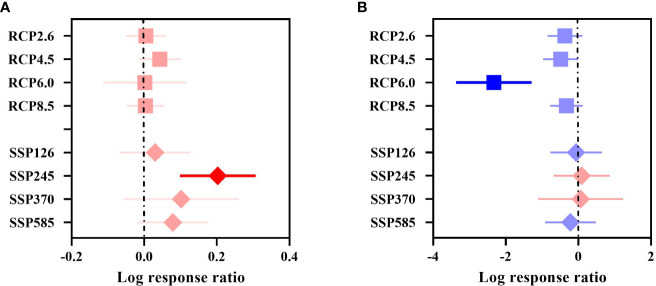
Effect sizes of different GHG concentrations. **(A)** indicates the overall suitable area, and **(B)** indicates the highly suitable area. The effects of different GHG concentrations on the future overall/highly suitable area (main effects) for woody plants under the RCP and SSP scenarios are indicated. Error lines indicate 95% confidence intervals. Positive effect (red color), significant positive effect (bright red color), negative effect (blue color), and significant negative effect (bright blue color).

The overall suitable area effect value QM= 14.2850, P = 0.0463 (P< 0.05), indicated that different future GHG concentrations had a significant effect on the overall change in the suitable area for woody plants in China, and all showed a positive response (**Figure 3A**). The increasing trend under the SSP scenario was consistent with that under the RCP scenario, reaching a significant level under SSP245, with an increase of 22.47%.

The highly suitable area effect value QM= 16.6432, P = 0.0198 (P< 0.05), indicated that different future GHG concentrations had a significant effect on the change in the highly suitable area for woody plants in China, with an overall decreasing trend in area under the RCP scenario (**Figure 3B**), with RCP4.5 (–38.57%) and RCP6.0 (–90.27%) reaching significant levels. None of the changes in the future highly suitable area under the SSP scenario were significant, with the largest decrease of –19.70% under SSP585.

In general, the overall suitable area under the different concentrations of GHGs in the RCP and SSP scenarios showed an increasing trend, and the overall suitable area increased by 1.58% under RCP and by 10.06% under SSP. The highly suitable area showed an overall decreasing trend, the highly suitable area under RCP decreased by 40.27%, and the highly suitable zone area under SSP decreased by 5.77%. The increase in the overall suitable area under the SSP scenario was larger than that under the RCP scenario, and the decrease in the highly suitable area under the SSP scenario was smaller than that under the RCP scenario.

### 3.5 Responses of different types of Chinese woody plants to future climate change

**Figure 4:**
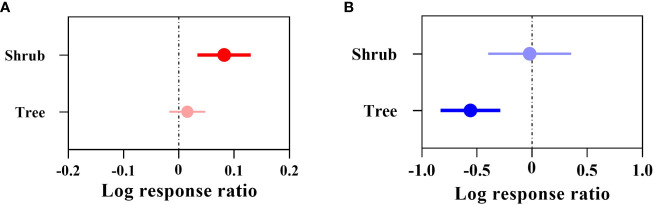
Effect sizes of the types of Chinese woody plants. **(A)** indicates the overall suitable area, and **(B)** indicates the highly suitable area. The effect of future climate on the overall/highly suitable area (main effects) for different woody plant types is indicated. Error lines indicate 95% confidence intervals. Positive effect (red color), significant positive effect (bright red color), negative effect (blue color), and significant negative effect (bright blue color).

The overall suitable area effect value QM (df = 1) = 5.0396, P = 0.0248 (P< 0.05) indicated that there was a significant difference in the change in overall suitable area for the different types of woody plants under future climate (**Figure 4A**), where the overall suitable area for shrubs (+8.52%) was significant in terms of its response to climate change. Among them, *Camellia petelotii*, *Lycium ruthenicum*, *Ammopiptanthus nanus*, etc., were the most affected. The overall suitable area for trees (+1.53%) was not significant. The highly suitable area effect value index QM (df=1) = 5.1427, P = 0.0233 (P< 0.05) indicated that there was a significant difference in the change in the highly suitable area for different types of woody plants under the future climate (**Figure 4B**), where the highly suitable area for trees will be significantly reduced by 42.81%. Among them, *Phoebe bournei*, *Ulmus pumila* and *Tsuga longibracteata* were the most affected. The highly suitable area for shrubs will be reduced by 2.20%, which did not reach the significance level.

In addition, the overall suitable area for both trees and shrubs showed an increasing trend, while the highly suitable area for both showed a decreasing trend.

### 3.6 Response of different Chinese woody plant genera to future climate change

**Figure 5:**
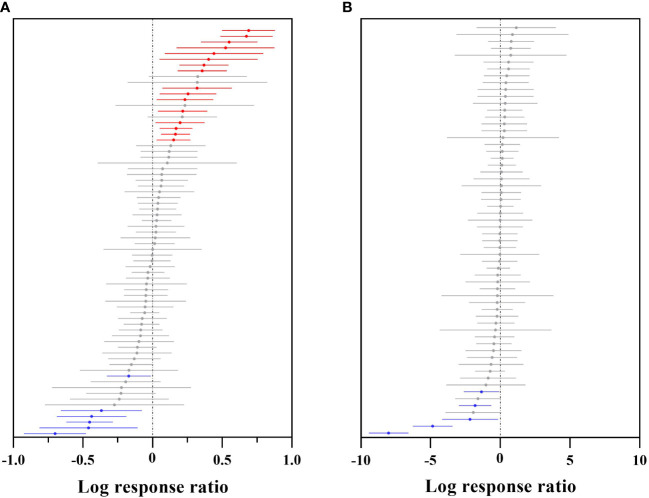
Effect sizes of different plant genera of Chinese woody plants. **(A)** indicates the overall suitable area, and **(B)** indicates the highly suitable area. The effects of future climate on the overall/highly suitable area (main effects) of different woody plant genera are indicated. Error lines indicate 95% confidence intervals. Significant positive effect (bright red color), significant negative effect (bright blue color), and nonsignificant effect (grey color).

The plants collected in this study that predicted an overall suitable area involved 71 genera, and those that predicted a highly suitable area involved 60 genera. The overall suitable area effect value QM (df = 70) = 357.0119, P< 0.0001 (P< 0.05), indicated significant differences in the overall suitable area changes for the different genera of woody plants under future climates (**Figure 5A** and **Supplementary Table 3**). A total of 33 genera had negative effects, among which 6 genera, including *Populus* (–50.40%) and *Brachychiton* (–36.88%), had significant negative effects, while 38 genera had positive effects, among which 16 genera, including *Dalbergia* (+99.21%), *Camellia* (+96.21%), and *Salix* (+73.29%), had significant positive effects.

The highly suitable area effect value QM (df = 59) = 189.6463, P< 0.0001 (P< 0.05), indicated that there was a significant difference in the change in the highly suitable area under future climates for the different genera of woody plants (**Figure 5B** and **Supplementary Table 3**). There were 33 genera with negative effects, including five genera with significant negative effects, such as *Ulmus* (–99.97%), *Phoebe* (–99.22%) and *Ostrya* (–88.61%); 27 genera had positive effects, among which *Cupressus*, *Fokienia*, and *Tetradium* more than doubled their future highly suitable areas, but none of the effects were significant.

Generally, the overall suitable areas for woody plants of different genera showed an increasing trend, with a few having a decreasing trend, while the highly suitable areas for the different genera showed a significant decreasing trend.

### 3.7 Response of Chinese woody plants in different floristic regions to future climate change

**Figure 6:**
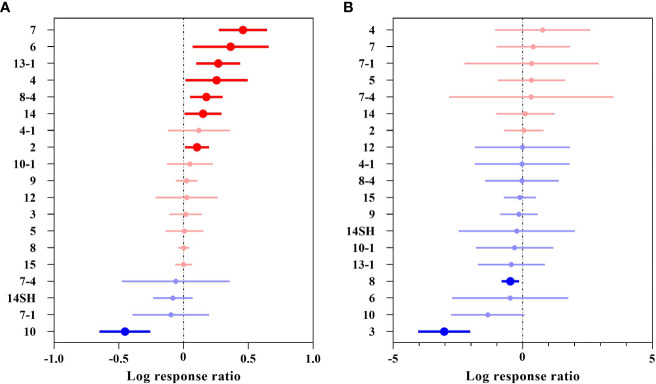
Effect sizes of different floristic regions of Chinese woody plants. **(A)** indicates the overall suitable area, and **(B)** indicates the highly suitable area. The effects of future climate on the overall/highly suitable area (main effects) for different woody plant floristic regions are indicated. Error lines indicate 95% confidence intervals. Positive effect (red color), significant positive effect (bright red color), negative effect (blue color), and significant negative effect (bright blue color).

The plants collected in this study were attributed to 19 different floristic regions, where the overall suitable area effect value, QM = 75.6180, P< 0.0001 (P< 0.05), indicated that woody plants from different floristic regions differed significantly in their overall suitable area changes under future climates (**Figure 6A** and **Supplementary Table 3**). There were 15 floristic regions with positive effects on area, among which Trop. Asia (Indo-Malesia), Trop. Asia to Trop. Africa and East C. Asia (or Asia Media), in Sinkiang (especially Kaschgaria), Kansu, Qinghai to Mongolia had significant positive effects, with 58.14%, 43.84% and 30.67% increases in their distribution areas, respectively. There were 4 floristic regions with negative effects, among which the Old World Temperate had a significant decrease of 36.40% in woody plants.

The highly suitable area effect value, QM= 36.8910, P = 0.0054 (P< 0.05), indicated that there was a significant difference in the change in the highly suitable area for the woody plants in the different floristic regions under future climates (**Figure 6B** and **Supplementary Table 3**), with a total of 12 floristic regions having a negative effect for woody plants, of which Trop. Asia & Trop. Amer. disjuncted and North Temperate had a significant negative effect with a decrease of 95.17% and a decrease of 37.77%, respectively. A total of 7 floristic regions had positive effects, but none of the effects were significant; the Old World Tropics had the largest increase at 116.63%.

Generally, the overall suitable area for woody plants in 15 floristic regions showed an increasing trend, and plants in 4 floristic regions showed a decreasing trend. The highly suitable area increased in only 7 floristic regions and decreased in 12 floristic regions, indicating that future climate change poses a greater threat to the highly suitable area for woody plants in different floristic regions.

## 4 Discussion

### 4.1 Impact of climatic variables on the future suitable area for woody plants

Climate change is multifaceted and includes changes in atmospheric GHG concentrations, temperature, and precipitation patterns, as well as increases in the frequency of extreme weather (Solomon et al., 2009; Gray and Brady, 2016; Pörtner et al., 2022), and changes in these factors have important implications for plant growth (Gutschick and BassiriRad, 2003; Bonan, 2008; Beer et al., 2010). According to projections, by 2100, East Asia will experience moderate summer warming (4-5°C), with continued increases in surface temperatures and increased precipitation in both summer and winter (Jiang et al., 2004; Feng et al., 2014; Liu, 2021). To quantify the impact of these changes on future changes in woody plant habitat area, this study examined the dominant climatic factors affecting changes in woody plant habitat area based on different climatic factors. BIO11 has a significant positive effect on both the overall and highly adaptable areas and is the main climatic factor for the future growth of woody plants. The change in temperature in winter has a great impact on the life activities of woody plants and is a limiting factor for the growth of woody plants distributed at middle altitudes (Jin et al., 2014). In addition, a number of other studies have yielded similar results; for example, using a newly compiled distribution map of 11422 woody plant species in eastern Eurasia, Su et al. (2020) estimated species richness patterns for all species and tropical and temperate relative families and found that winter temperature was the best predictor of woody plant richness patterns, and Wang et al. (2011) modelled the distribution of woody plants in China and predicted the species richness of woody plants in North America and the Northern Hemisphere, showing that the mean temperature of the coldest quarter was the strongest predictor of species richness. When the temperature is warmer in winter, the physiological activities of trees are strengthened (Janecka et al., 2016). Warm autumn and winter can increase the storage of photosynthetic products in the next year. It will promote the radial growth of woody plants at low and medium altitudes (Yang, 2020). For the overall suitable area, BIO03 has a significant positive effect, and BIO02 has a significant negative effect. Tree growth mainly occurs at night, while photosynthesis and transpiration occur in the daytime, which indicates that diurnal temperature ranges and atmospheric water demand are critical to the growth response (Zweifel et al., 2021). Daytime warming can reduce tree growth (Tao et al., 2022); when the diurnal temperature ranges are small, the net growth of trees is large (Zhang et al., 2022). Therefore, higher BIO2 is not conducive to plant growth, and appropriate BIO3 is conducive to the expansion of the plant habitat. For highly suitable areas, future extreme changes in BIO15 and BIO04 will be beyond the tolerance range of woody plants (Jiang et al., 2004), and this is also the main factor leading to the decline in future highly suitable areas for woody plants in China. The plant height suitable area is more strict to the site conditions, and the high temperature in summer will shorten the leaves (Schoettle, 1990), which is not conducive to plant photosynthesis and easily causes plant water deficit. The low temperature in winter will cause freezing damage or cold damage to plants and may also interfere with the water relationship of trees (López-Bernal et al., 2015). In particular, evergreen broad-leaved trees from warm climates are more vulnerable to winter low temperatures(Jalili et al., 2010). The increase in precision will reduce the stability effect intensity of biodiversity of the community (the strength of the stabilizing effects of biodiversity) (Liang et al., 2022). It is not conducive to the stability of woody plant communities. Therefore, BIO04, BIO11 and BIO15 have an important impact on the highly adaptable area of woody plants, which may be related to the future extreme climate frequency (Breshears et al., 2005; Royer et al., 2011; Lloret et al., 2012). At the same time, climate change has a direct binding effect on species ranges (Feng et al., 2019). Plant species respond to the effects of climate change primarily through population movements and range changes (Svenning et al., 2015). Different plants are able to respond differently to environmental (e.g., climatic) stresses due to differences in their morphological and physiological attributes (Shmida and Burgess, 1988). The majority of woody plants in the raw data selected for this study moved to high latitudes (e.g., *Litsea, Nothotsuga, Picea*), with only a small number of species moving to lower latitudes or remaining unchanged in their distribution (e.g., *Ostrya*, *Phoebe*). Peng et al. (2022) also found that woody plant species richness declines more in southern China than in the north under future climate change.

Overall, the mean temperature of the coldest quarter is the main factor affecting the future habitat area of woody plants (Wang et al., 2011; Hatfield and Prueger, 2015; Wu et al., 2015), and it also has an impact on the migration of most woody plants (Root et al., 2003). Moreover, temperature fluctuations also have an important impact on the area of potential suitable areas for woody plants in the future.

### 4.2 Effects of greenhouse gas concentration pathways on the future habitat of woody plants

Our study shows that the change in future woody plant suitable areas under the SSP scenario was more subtle than that under the RCP scenario, perhaps because the SSP scenario adds different social factors and measures for future climate change mitigation (Lauri et al., 2019; Wu et al., 2019; Meinshausen et al., 2020). Under the different GHG concentrations in the RCP and SSP scenarios, the overall suitable habitat area for woody plants increased, but the highly suitable area decreased. The magnitude of GHG concentrations under the different emission scenarios did not show a significant linear relationship with the future changes in woody plant area, probably because of the large differences among different woody plants. Increased atmospheric CO_2_ concentrations can increase photosynthetic rates, and the higher the temperature is within a certain range, the faster the photosynthetic rate; in addition, moderate climate warming is conducive to species dispersal and population size increases (Bazzaz, 1990; Thomas et al., 2004; Schöb et al., 2009). As CO_2_ concentrations and temperatures continue to increase, soil water starts to evaporate, and the photosynthetic rates of woody plants gradually decrease (Oren et al., 2001; Ainsworth and Long, 2005; Hyvönen et al., 2007; Meier et al., 2012; Wu et al., 2015). Therefore, in the future, a large amount of highly suitable area for woody plants in China will be converted to moderate or minimally suitable region areas, resulting in a loss of highly suitable areas of up to 42.81%. In contrast, woody plants are tolerant to adversity, and thus, temporarily, there will be less impact on the overall suitable region area, which, together with the conversion from highly fit areas, will even increase by 3.6552%.

In the RCP scenario, the highly adaptable area of RCP6.0 decreases the most, while the highly adaptable area of RCP8.5 decreases slightly, but the highly adaptable area of most species decreases, and only the highly adaptable area of a few species increases significantly, which is not conducive to maintaining the species diversity of woody plants, thus affecting population stability (Liang et al., 2022). In the SSP scenario, the highly adaptable area of SSP585 decreased the most, the overall highly adaptable area of SSP245 increased more, and the highly adaptable area also increased slightly. This may be because climate warming promotes species transfer to high latitudes and altitudes, especially those from cold temperate zones (Calef et al., 2005; Jalili et al., 2010). In general, under the low emission scenario, the impact on the highly adaptable area is small, especially in SSP245, and both the overall adaptable area and the highly adaptable area have increased, indicating that moderate warming is beneficial to the expansion of the overall area of woody plants, but under the high concentration emission scenario, the fragmentation of species habitat may be intensified, which will have a negative impact on the woody plant population. This is similar to the findings of Inague et al. (2021) and others, where the biodiversity of Brazilian woody plants will continue to be lost with the gradual increase in greenhouse gas emission concentrations.

### 4.3 Differences in the properties of woody plants under the influence of climate change

Woody plants with longer life cycles and larger body sizes are more tolerant to harsh conditions such as habitat fragmentation or climate change (Harper, 1977; Petit and Hampe, 2006; Sork et al., 2013; Olson et al., 2016; Llanes et al., 2021). Because shrubs have a shorter life cycle and can settle in new suitable areas more quickly than trees, the overall suitable area for shrubs (+8.52%) increased more, and the highly suitable area for shrubs (–0.22%) decreased less than that of trees (–42.81%) under future climate change projections (Hiernaux et al., 2009). The results of relevant remote sensing, sample plot surveys and tree ring research also show that climate warming has led to significant growth acceleration and expansion of global shrub vegetation (Screen, 2014). The results that the number of shrub species was greater than the number of tree species with the increase in overall suitable area and the number of tree species was greater than the number of shrub species with the decrease in highly suitable area are further evidence that shrubs are more responsive to climate than trees. At the same time, according to the results of Zhang et al. (2018), from 1999-2014, China’s provincial carbon emission efficiency, forestland carbon sinks accounted for >90% of the total carbon sinks, so forest ecosystems play an important role as carbon sinks. Our results suggest that the overall area of suitable habitat for both trees and shrubs will increase in the future, which offers the potential for future expansion of planted forests, but a significant decrease in the area of highly suitable habitat for trees will also be an important challenge for the carbon sink capacity of forest ecosystems.

By quantifying the trends in the response of different woody plant genera to climate change, it was found that their responses vary greatly (Dawson et al., 2011). The future climate warming, the reduction in precipitation and the increase in potential evaporation mean an increase in drought frequency and intensity (Su et al., 2021a), which will lead to a large expansion of drought-tolerant plants (e.g., *Dalbergia*, *Cupressus*, and *Xanthoceras*) and some plants that are highly adaptable (e.g., *Camellia, Cassia*, and *Fokienia*), with extreme increases in *Fokienia* mainly due to its expansion in places suitable for relic plants (Chen et al., 2019; Liu et al., 2022). Woody plants that cannot adapt to climate change (e.g., *Populus*, *Hippophae*, and *Nothotsuga*) will decline dramatically and are at high risk of extinction, and more attention should be given to these genera as part of future conservation efforts. In addition, the reduction in species diversity will lead to a change in the species composition of the community, resulting in a gradual turnover of species in the community to more drought-tolerant species (Root et al., 2003; Hiernaux et al., 2009). In carbon sinks, the habitat area of plants with large carbon sink benefits, such as *Salix* (Gong, 2019), *Cupressus* (Yao et al., 2014; Ma et al., 2022) and *Camellia* (Zhang et al., 2017), will increase significantly, which will make a larger contribution to the future forest carbon sink system in China. However, the habitat area of high-quality carbon sink plant species such as *Populus* (Fang et al., 2007), *Taiwania* (Xie et al., 2020) and *Phoebe* (Ma et al., 2009) will face a large decrease, so this is an area of concern for future forestry management authorities when they formulate relevant policies in response to climate change in the future.

For woody plants from different floristic regions, the overall and highly suitable areas in the tropical region generally showed an increasing trend, indicating that climate warming will have fewer negative effects on future area changes of plants in the tropical region, perhaps because tropical vegetation is more resilient to climate warming disturbances and can reduce the effects by altering its adaptation mechanisms (Bazzaz, 1990; Borchert, 1998; Wang et al., 2011; Ciemer et al., 2019). Meanwhile, other studies have shown that woody plant diversity is mainly due to an increase in the intensity of frost filtration of tropical species from the equator/lowlands to the poles/uplands, with the abundance of tropical plants decreasing more rapidly than temperate plants as latitude increases and the average temperature of the coldest season decreases(Wang et al., 2011; Shiono et al., 2018). However, as the temperature rises, the previously colder regions can gradually allow the tropical flora to survive. It should be noted that the overall suitable area of (3) had a weak increasing trend (1.66%), but the highly suitable area decreased by 95.17%; however, (10) (the overall suitable area –36.40%, the highly suitable area –73.98%) and (14SH) (the overall suitable area –7.95%, the highly suitable area –20.24%) showed a decreasing trend. Therefore, in future woody plant conservation, these three floristic regions should be prioritized for conservation.

## 5 Conclusion

Future climate change will result in a 3.66% increase in the overall suitable areas and a 31.33% decrease in the highly suitable areas in China. The mean temperature of the coldest quarter had a significant positive effect on both the overall suitable areas and the highly suitable areas. The effects of different GHG concentrations in the SSP scenarios on woody plants were more moderate than those in the RCP scenarios. Moderate warming is conducive to the expansion of the overall areas of woody plants. However, high emission concentrations will increase the fragmentation of suitable woody plant areas and reduce species diversity in the future. Future extreme weather events may pose a great threat to the survival of woody plants. At the same time, we should also focus on the conservation of *Populus*, *Hippophae*, and *Nothotsuga* and woody plant conservation in floristic regions 3, 10, and 14SH. However, since this study mainly focused on the response of woody plant growth to climatic factors, it does not further quantify the effects of human activities (e.g., urbanization and afforestation area), species characteristics (e.g., pollen dispersal ability, interspecific competition), and external factors (e.g., terrain and soil) on the changes in woody plant suitable areas, which may lead to an overestimation of the predicted habitat areas. Further nonclimatic factors can be added in future studies, and attention needs to be paid to the synergistic effects between different pressures to more accurately reveal the future distribution patterns and area changes of woody plants.

## Data availability statement

The original contributions presented in the study are included in the article/**Supplementary Material**. Further inquiries can be directed to the corresponding author.

## Author contributions

Conceptualization, PT, YL; Methodology, PT; Data curation, PT, YL; Formal analysis, YL; Data collection, PT, YL; Project administration, YL; Software, PT and YL, Supervision, JO; Writing – original draft, PT; Writing – review and editing, PT, YL. All authors contributed to the article and approved the submitted version.
