# Supplementary material for: Meta-analysis of the impact of future climate change on the area of woody plant habitats in China
Source: Front Plant Sci. 2023 Mar 15;14:1139739. doi: 10.3389/fpls.2023.1139739 (PMC10050603; doi:10.3389/fpls.2023.1139739)
Supplement: Supplementary file 1 [file DataSheet_1.docx]

Supplementary Material

# Supplementary Data

Supplementary Data have been uploaded separately on submission.

# Data sources. List of articles used in the review and meta-analysis.

Chen, A., Gong, W., Kong, F., Zhao, Z., Tao, jun, 2021. Predicting the influence of climate change on the suitable range for planting *Myrica rubra*. Journal of Yangzhou University 42, 96–102. https://doi.org/10.16872/j.cnki.1671-4652.2021.04.017

Chen, Y., Lu, S., Mao, L., 2021. Prediction of future changes in suitable distribution area for rare tree species of *Dalbergia*. Journal of Zhejiang A&F University 38, 837–845. https://doi.org/10.11833/j.issn.2095-0756.20200522

Chen, Y., Lv, Y., Yin, X., 2019. Predicting habitat suitability of 12 coniferous forest tree species in southwest China based on climate change. Journal of Nanjing Forestry University 43, 113–120. https://doi.org/10.3969/j.issn.1000-2006.201808045

Chen, Yuguang, Le, X., Chen, Yuhan, Cheng, W., Du, J., Zhong, Q., Cheng, D., 2022. Identification of potential distribution area of *Cunninghamia lanceolata* in China under climate change based on the MaxEnt model. Chinese Journal of Applied Ecology 33, 1207–1214. https://doi.org/10.13287/j.1001-9332.202205.024

Cui, J., 2015. Potential impacts of climate change on three commonly used medicinal plants (Master’s Thesis). Shaanxi Normal University.

Dong, J., Chen, J., Yang, H., Li, M., Wang, X., Yi, X., 2022. Simulation and analysis of ecological characteristics of *Cerasus conradinae* adaptability area. Journal of Nanjing Forestry University 46, 213–221. https://doi.org/10.12302/j.issn.1000-2006.202101024

Du, Z., He, Y., Wang, H., Wang, C., Duan, Y., 2021. Potential geographical distribution and habitat shift of the genus *Ammopiptanthus* in China under current and future climate change based on the MaxEnt model. Journal of Arid Environments 184, 104328. https://doi.org/10.1016/j.jaridenv.2020.104328

Fan, X., Pan, J., He, S., 2021. Prediction of the Potential Distribution of *Rosa roxburghii* under the Background of Climate Change Based on MaxEnt Model. Acta Bot,Boreal, Occident.Sin. 41, 159–167. https://doi.org/10.7606/j.issn.1000-4025.2021.01.0159

Guo, W., Hao, L., Zhang, G., Huang, H., Ning, J., Li, Y., 2020. Potential Geographic Distribution of *Salix psammophila* based on MaxEnt. Journal of Inner Mongolia Forestry Science & Technology 46, 1–7. https://doi.org/10.3969/j.issn.1007-4066.2020.03.001

Han, S., Yan, W., Yang, X., Hu, B., Yu, F., 2022. Potential distribution pattern and future change of *Ulmus pumila* in China based on MaxEnt model. Journal of Nanjing Forestry University 1–9.

He, X., Ma, W., Zhao, T., Ma, Q., Liang, L., Wang, G., Yang, Z., 2022. Prediction of Potential Distribution of Endangered Species *Corylus chinensis* Franch. in Climate Change Context. Forest Research 35, 104–114. https://doi.org/10.13275/j.cnki.lykxyj.2022.01.012

He, X., Ning, X., Guo, Y., Wei, H., 2019. Geographical distribution of  *Xanthoceras sorbifolia*  Bunge in China and predicting suitable area under the climate change scenario. Research of Agricultural Modernization 40, 316–324. https://doi.org/10.13872/j.1000-0275.2019.0022

He, X., Si, J., Zhao, C., Wang, C., Zhou, D., 2021. Potential distribution of *Hippophae thibetana* and its predicted responses to climate change. Journal of Desert Research 41, 101–109. https://doi.org/10.7522/j.issn.1000-694X.2021.00028

Hu W., Zhang Z.-Y., Chen L.-D., Peng Y.-S., Wang X., ^1^Lushan Botanical Garden, Chinese Academy of Sciences, Provincial Key Laboratory of Plant ex Situ Conservation and Utilization, Jiujiang, Jiangxi 332900, China^2^Forestry Industry Development Administration of Jiangxi Province, Nanchang 330006, China, 2020. Changes in potential geographical distribution of *Tsoongiodendron odorum* since the Last Glacial Maximum. Chinese Journal of Plant Ecology 44, 44–55. https://doi.org/10.17521/cjpe.2018.0258

Huang, J., 2020. Priority Analyses on Introduction of Five Exotic Garden Tree in China under Climate Change (Master’s Thesis). Zhongkai University of Agriculture and Engineering.

Jian, S., Zhu, T., Wang, J., Yan, D., 2022. The Current and Future Potential Geographical Distribution and Evolution Process of *Catalpa bungei* in China. Forests 13, 96. https://doi.org/10.3390/f13010096

Li, J., 2021. Geographical distribution and suitabilitycultivation area of five typical ecological treespecies on the QingHai-tibet plateau (Master’s Thesis). Tibet University.

Li, N., Zhang, A., Zhang, L., Wanag, K., Luo, H., Pan, K., 2019. Predicting Potential Distribution of Two Species of Spruce in Qinghai-Tibet Plateau under Climate Change. Bulletin of Botanical Ｒesearch 39, 395–406. https://doi.org/10.7525 /j.issn.1673-5102.2019.03.010

Li, Y., Jiang, X., Deng, M., Li, Q., 2017. Potential distribution modeling and analysis of *Quercus phillyraeoides.* Chinese Journal of Ecology 36, 2971–2978. https://doi.org/10.13292/j.1000-4890.201710.036

Li, Y., Li, M., Li, C., Liu, Z., 2020. Optimized Maxent Model Predictions of Climate Change Impacts on the Suitable Distribution of *Cunninghamia lanceolata* in China. Forests 11, 302. https://doi.org/10.3390/f11030302

Li, Y., Zhang, C., Zhu, H., Li, X., Duan, Y., Wang, X., 2019. Analyses on suitable distribution areas and main climatic variables of  *Osmanthus yunnanensis* and  *O. delavayi*. Journal of Plant Resources and Environment 28, 71–78. https://doi.org/10.3969/j.issn.1674-7895.2019.01.09

Li Y., Zhang L., Zhu W., Zhang J., Xu S., Zhu L., 2021. Changes of Taxus chinensis var. mairei habitat distribution under global climate change. Journal of Natural Resources 36, 783–792. https://doi.org/10.31497/zrzyxb.20210318

Liu, T., Cao, J., Qi, R., Li, B., Gao, B., 2022. Research of Potential Geographical Distribution of *Picea pur purea*  Based on GIS and MaxEnt under Different Climate Conditions. Acta Bot,Boreal, Occident.Sin. 42, 481–491. https://doi.org/10.7606/j. issn.1000-4025.2022.03.0481

Liu, W., Zhao, R., Geng, X., Zhu, Z., 2021. Geographical distribution and potential distribution area prediction of *Paeonia jishanensis* in China. Journal of Beijing Forestry University 43, 83–92. https://doi.org/10.12171/j.1000-1522.20200360

Liu, Z., Hu, L., 2022. Prediction of Potential Distribution and Climate Change of Ｒare Species *Cephalotaxus oliveri*. Forest Resources Management 35–42. https://doi.org/10.13466/j.cnki.lyzygl.2022.01.005

Lu, K., He, Y., Mao, W., Du, Z., Wang, L., Liu, G., Feng, W., Duan, Y., 2020. Potential geographical distribution and changes of  *Artemisia ordosica* in China under future climate change. Chinese Journal of Applied Ecology 31, 3758－3766. https://doi.org/10.13287/j.1001-9332.202011.017

Meng, Y., 2019. Genetic Diversity Analysis of Disanthus *cercidifolius* var. *longipes* (Master’s Thesis). Central South University of Forestry and Technology.

Ning, H., Ling, L., Sun, X., Kang, X., Chen, H., 2021. Predicting the future redistribution of Chinese white pine *Pinus armandii* Franch. Under climate change scenarios in China using species distribution models. Global Ecology and Conservation 25, e01420. https://doi.org/10.1016/j.gecco.2020.e01420

Pan, J., Fan, X., Luo, S., Zhang, Y., Yao, S., Guo, Q., Qian, Z., 2020. Predicting the Potential Distribution of Two Varieties of *Litsea coreana* (Leopard-Skin Camphor) in China under Climate Change. Forests 11, 1159. https://doi.org/10.3390/f11111159

Pan, T., 2021. The phylogeographic structure and climate response characteristics of three spring blossom *Osmanthus* species (Master’s Thesis). Nanjing Forestry University.

Qin Y., Lu K., Du Z., Shi J., Chai G., Zhang Y., Lei K., Duan Y., 2022. Potential changes in the geographical distribution of the relict plant *Potaninia mongolica* Maxim. in China under climate change scenarios. Acta Ecologica Sinica 42, 1–12. https://doi.org/10.5846/stxb202106111553

Qiu H., Sun J., Xu D., Jiao J., Xue M., Yuan W., Shen A., Jiang B., Li S., 2020. The distribution dynamics of *Ormosia hosiei* under different climate change scenarios since the Last Glacial Maximum. Acta Ecologica Sinica 40, 3016–3026. https://doi.org/10.5846/stxb201904080688

Qiu, H., Sun, J., Xu, D., Shen, A., Jiang, B., Yuan, W., Li, S., 2022. MaxEnt model-based prediction of potential distribution of *Liriodendron chinense* in China. Journal of Zhejiang A&F University 37, 1–8. https://doi.org/10.11833/j.issn.2095-0756.2020.01.001

Ran Q., Wei H., Zhao Z., Zhang Q., Liu J., Gu W., 2019. Impact of climate change on the potential distribution and habitat fragmentation of the relict plant *Cathaya argyrophylla* Chun et Kuang. Acta Ecologica Sinica 39, 2481–2493. https://doi.org/10.5846/stxb201802010282

Sayit, H., Nurbay, A., Li, X., Shao, H., Arman, J., Ateng, G., 2018. Effects of Climate Change and Human Activities on the Distribution Pattern of *Calligonum mongolicum* Turcz. ARID ZONE RESEARCH 35, 1450–1458. https://doi.org/10.13866/j.azr.2018.06.23

Shen, J., Chen, D., Hong, Y., Sun, X., Zhang, S., 2019. Prediction on potential distribution areas of *Larix kaempferi*  in China based on MaxEnt model. Journal of Plant Resources and Environment 28, 19–25. https://doi.org/10.3969/j.issn.1674-7895.2019.03.03

Song, Y., Zhang, G., Jia, Q., Wen, Q., Mo, X., Liu, L., 2021. Prediction of Potential Distribution of *Ormosia henryi* in China Under Climate Change. Journal of Northwest Forestry University 36, 108–115. https://doi.org/10.3969/j.issn.1001-7461.2021.06.15

Sun, J., Qiu, H., Guo, J., Xu, X., Wu, D., Zhong, L., Jiang, B., Jiao, J., Yuan, W., Huang, Y., Shen, A., Wang, W., 2020. Modeling the potential distribution of *Zelkova schneideriana* under different human activity intensities and climate change patterns in China. Global Ecology and Conservation 21, e00840. https://doi.org/10.1016/j.gecco.2019.e00840

Tan X., Zhang L., Zhang A., Wang Y., Huang D., Wu X., Sun X., Xiong Q., Pan K., 2018. The suitable distribution area of *Tsuga longibracteata* revealed by a climate and spatial constraint model under future climate change scenarios. Acta Ecologica Sinica 38, 8934–8945. https://doi.org/10.5846/stxb201806021236

Tang, S.-L., Song, Y.-B., Zeng, B., Dong, M., 2022. Potential distribution of the extremely endangered species *Ostrya rehderiana* (Betulaceae) in China under future climate change. Environ Sci Pollut Res 29, 7782–7792. https://doi.org/10.1007/s11356-021-16268-1

Tang, X., Yuan, Y., Zhang, J., 2021. Simulation of Potential Spatial-temporal Population Dynamics of*Pinus tabuliformis* under Climate Change. Journal of Northeast Forestry University 49, 1–7. https://doi.org/10.13759/j.cnki.dlxb.2021.09.001

Tang, Y., Zhao, R., Ren, G., Cao, F., Zhu, Z., 2021. Prediction of potential distribution of *Lycium chinense* based on MaxEnt model and analysis of its important influencing factors. Journal of Beijing Forestry University 43, 23–32. https://doi.org/10.12171/j.1000−1522.20200103

Tiamiyu, B.B., Ngarega, B.K., Zhang, X., Zhang, H., Kuang, T., Huang, G.-Y., Deng, T., Wang, H., 2021. Estimating the Potential Impacts of Climate Change on the Spatial Distribution of *Garuga forrestii*, an Endemic Species in China. Forests 12, 1708. https://doi.org/10.3390/f12121708

Wan, G., Shen, L., Guo, Y., Jin, L., Chen, J., 2021. Potential Geographical Distribution of *Forsythia Suspensa* (Thunb.) Vahl under ClimateChange Based on Maximum Entropy Model. Chin J Inf Tradit Chin Med 28, 1–6. https://doi.org/10.19879/j.cnki.1005-5304.202105437

Wang, Q., Fan, B., Zhao, G., 2002. Prediction of potential distribution area of *Corylus mandshurica* in China under climate change. Chinese Journal of Ecology 39, 3774–3784. https://doi.org/10.13292/j.1000-4890.202011.014

Wang, X., Liu, G., Xiao, T., 2022. Suitability Characteristics of *Camellia oleifera* Growth under Climate Change Scenarios. Tropical Geography 40, 868–880. https://doi.org/10.13284/j.cnki.rddl.003267

Wen, G., Ye, X., Lai, W., Shi, C., Huang, Q., Ye, L., Zhang, G., 2021. Dynamic analysis of mixed forest species under climate change scenarios. Ecological Indicators 133, 108350. https://doi.org/10.1016/j.ecolind.2021.108350

Wen, G., YE, X., Shi, C., Lai, W., Liu, B., Jiang, T., Zhu, X., Zhang, G., 2022. Potential suitable area of *Altingia multinervis* predicted by optimizated MaxEnt model. Guihaia 42, 363–372. https://doi.org/10.11931/guihaia.gxzw202103041

Wu, F., Zhu, P., Ji, K., 2022. Responses of masson pine( *Pinus massoniana*) distribution patterns to future climate change. Journal of Nanjing Forestry University 46, 196–204. https://doi.org/10.12302/j.issn.1000-2006.202008019

Wu, X., Ye, D., Bai, Y., Duan, G., Wang, L., Sun, H., 2022. Distribution Pattern and Future Change of *Picea meyeri* in China Based on MaxEnt Model. Acta Bot,Boreal, Occident.Sin. 42, 162–172. https://doi.org/10. 7606/j.issn.1000-4025.2022.01.0162

Wu, Y.-M., Shen, X.-L., Tong, L., Lei, F.-W., Mu, X.-Y., Zhang, Z.-X., 2021. Impact of Past and Future Climate Change on the Potential Distribution of an Endangered Montane Shrub *Lonicera oblata* and Its Conservation Implications. Forests 12, 125. https://doi.org/10.3390/f12020125

Xiao J., Ding X., Cai C., Zhang C., Zhang X., Li L., Li J., 2021. Simulation of the potential distribution of *Phoebe bournei*  with climate changes using the maximum-entropy ( MaxEnt) model. Acta Ecologica Sinica 41, 5703–5712.

Xie, C., Huang, B., Jim, C.Y., Han, W., Liu, D., 2021. Predicting differential habitat suitability of *Rhodomyrtus tomentosa* under current and future climate scenarios in China. Forest Ecology and Management 501, 119696. https://doi.org/10.1016/j.foreco.2021.119696

Xiong, Z., Zhang, X., Zou, X., Zhao, Y., Chen, X., 2019. Prediction of the suitable distribution and responses to climate change of *Sorbus tianschanica* in China. Ecological Science 38, 44–51. https://doi.org/10.14108/j.cnki.1008-8873.2019.05.007

Xu, X., Zhang, H., Xie, T., Xu, Y., Zhao, L., Tian, W., 2017. Effects of Climate Change on the Potentially Suitable Climatic Geographical Range of *Liriodendron chinense*. Forests 8, 399. https://doi.org/10.3390/f8100399

Xu, Y., 2019. Predicting the Suitable Habitats of Relic Plants *Ginkgo biloba* and *Davidia involucrata* (Master’s Thesis). North China Electric Power University.

Yan, X., Wang, S., Duan, Y., Han, J., Huang, D., Zhou, J., 2021. Current and future distribution of the deciduous shrub *Hydrangea macrophylla* in China estimated by MaxEnt. Ecology and Evolution 11, 16099–16112. https://doi.org/10.1002/ece3.8288

Ye, L., Zhang, W., Ye, X., Liu, Y., Zhang, G., Liu, B., Ruan, S., 2021. Prediction of Potential Distribution Area and Analysis of DominantEnvironmental Variables of *Davidia involucrate* Based on Maxent. Journal of Sichuan Agricultural University 39, 604–612. https://doi.org/10.16036/j. issn.1000-2650.2021.05.006

Ye, X., Chen, F., Sun, R., Wu, N., Liu, B., Song, Y., 2019. Prediction of Potential Suitable Distribution Areas for *Choerospondias axillaris* besed on MaxEnt Model. Acta Agriculturae Universitatis Jiangxiensis 41, 440–446. https://doi.org/10.13836/j.jjau.2019052

Ye, X., Zhang, M., Lai, W., Yang, M., Fan, H., Zhang, G., Chen, S., Liu, B., 2021. Prediction of potential suitable distribution of *Phoebe bournei* based on MaxEnt optimization model. Acta Ecologica Sinica 41, 8135–8144. https://doi.org/10.5846/stxb202007131822

Ye, X., Zhao, G., Zhang, M., Cui, X., Fan, H., Liu, B., 2020. Distribution Pattern of Endangered Plant *Semiliquidambar cathayensis* (Hamamelidaceae) in Response to Climate Change after the Last Interglacial Period. Forests 11, 434. https://doi.org/10.3390/f11040434

Zeng, M., 2021. Study on the distribution and potential suitable area of rare and endangered plant *Cercidiphyllum japonicum* (Master’s Thesis). China West Normol University.

Zhai, X., Shen, Y., Zhu, S., Tu, Z., Zhang, C., Li, H., 2021. Potential Impacts of Climate Change in Future on the Geographical Distributions of Relic  *Liriodendron chinense*. Journal of Tropical and Subtropical Botany 29, 151–161. https://doi.org/10.11926/jtsb.4322

Zhang, C., He, J., Sun, Y., Li, K., 2018. Distributional Change in Suitable Areas for *T. ciliata*  var.  *pubescens*  Based on MaxEnt. Forest Ｒesearch 31, 120–126. https://doi.org/10.13275/j.cnki. lykxyj.2018.03.016

Zhang C., He J., Sun Y., Li K., 2018. Prediction of distributional change of *Toona ciliata*  var.  *ciliate* and application in regionalization of introduction based on MaxEnt. Journal of Yunnan University 40, 164–173. https://doi.org/10.7540/j.ynu.20170013

Zhang, C., He, J., Sun, Y., Li, K., 2017. Distributional change in suitable areas for *Toona sureni* based on MaxEnt model．. Journal of Beijing Forestry University 39, 33–41. https://doi.org/10.13332/j.1000-1522.20170002

Zhang, H., Zhao, H., Xu, C., 2021. The potential geographical distribution of *Alsophila spinulosain* under climate change in China. Chinese Journal of Ecology 40, 968–979. https://doi.org/10.13292/j.1000-4890.202104.022

Zhang, J.-H., Li, K.-J., Liu, X.-F., Yang, L., Shen, S.-K., 2021. Interspecific Variance of Suitable Habitat Changes for Four Alpine *Rhododendron* Species under Climate Change: Implications for Their Reintroductions. Forests 12, 1520. https://doi.org/10.3390/f12111520

Zhang, K., Zhang, Y., Jia, D., Tao, J., 2020. Species Distribution Modeling of *Sassafras Tzumu* and Implications for Forest Management. Sustainability 12, 4132. https://doi.org/10.3390/su12104132

Zhang, L., Wei, Y., Wang, J., Zhou, Q., Liu, Fenggui, Chen, Q., Liu, Fei, 2020. The potential geographical distribution of *Lycium ruthenicum* Murr under different climate change scenarios. HIN J APPL ENVIRON BIOL 26, 969–978. https://doi.org/10.19675/j.cnki.1006-687x.2019.09046

Zhang, L., Zhu, L., Li, Y., Zhu, W., Chen, Y., 2022. Maxent Modelling Predicts a Shift in Suitable Habitats of a Subtropical Evergreen Tree (*Cyclobalanopsis glauca* (Thunberg) Oersted) under Climate Change Scenarios in China. Forests 13, 126. https://doi.org/10.3390/f13010126

Zhang, M., Ye, X., Li, J., Liu, Y., Chen, S., Liu, B., 2021. Prediction of potential suitable area of *Ulmus elongata* in China under climate change scenarios. Chinese Journal of Ecology 40, 3822–3835. https://doi.org/10.13292/j.1000-4890.202112.018

Zhang, M., Ye, X., Liu, Y., Li, J., Chen, S., Zhang, G., Liu, B., 2022. Predicting the potential geographical distribution of *Erythrophleum fordiiin* China based on SSPs. Journal of Beijing Forestry University 44, 54–65. https://doi.org/10.12171/j.1000−1522.20210308

Zhang, X., Chen, C., Gao, F., Yuan, S., Han, S., Ni, Z., Yu, J., 2022. Spatial distribution of *Larix gmelinii* forests in Northeast China and its response to climate change. Chinese Journal of Ecology 44, 1041–1049. https://doi.org/10.13292/j.1000-4890.202205.009

Zhang, X., Deng, T., Luo, L., Li, J., 2021. Prediction of Potential Suitable Area of *Rosa persica* and Study on Its Vulnerable Mechanism. Acta Bot. Boreal.-Occident. Sin. 9, 1570–1582. https://doi.org/10.7606/j.issn.1000-4025.2021.09.1570

Zhang, X., Li, Y., Xie, Y., Bao, X., Fang, Y., 2018. Effect of climate change on potential geographical distribution of Sorbus amabilis. Journal of Plant Resources and Environment 27, 31–41. https://doi.org/10.3969/j.issn.1674-7895.2018.04.04

Zhao, H., 2021. Response of potential geographical distribution of eight China’s first-class rare and endangered plants to climate change and analysis of GAP (Master’s Thesis). Northwest Normal University.

Zhao, H., Zhang, H., Xu, C., 2020. Study on *Taiwania cryptomerioides* under climate change: MaxEnt modeling for predicting the potential geographical distribution. Global Ecology and Conservation 24, e01313. https://doi.org/10.1016/j.gecco.2020.e01313

Zhao, Q., Mi, Z., Lu, C., Zhang, X., Chen, L., Wang, S., Niu, J., Wang, Z., 2022. Predicting potential distribution of *Ziziphus spinosa* (Bunge) H.H. Hu ex F.H. Chen in China under climate change scenarios. Ecology and Evolution 12. https://doi.org/10.1002/ece3.8629

Zhao, R., He, Q., Chu, X., Lu, Z., Zhu, Z., 2019. Prediction of potential distribution of *Carpinus cordata* in China under climate change. Chinese Journal of Applied Ecology 30, 3833–3843. https://doi.org/10.13287/j.1001-9332.201911.020

Zhao, Y., Deng, X., Xiang, W., Chen, L., Ouyang, S., 2021. Predicting potential suitable habitats of Chinese fir under current and future climatic scenarios based on Maxent model. Ecological Informatics 64, 101393. https://doi.org/10.1016/j.ecoinf.2021.101393

Zhao, Z., Wei, H., Guo, Y., Zhao, Zebin, Pang, G., Ma, Y., Gu, W., 2019. Impacts of Climate Change on Cultivation Suitability of *Lycium ruthenicum*. Journal of Desert Research 37, 902–908. https://doi.org/10.14108/j.cnki.1008-8873.2019.05.007

Zheng, W., Cao, K., 2019. Potential geographical distribution of five *Lithocarpus* species in China and their response to climate change. Plant Science Journal 37, 474–484. https://doi.org/10.11913/PSJ. 2095-0837.2019.40474

Zhou, X., 2021. Molecular Identification of *Fimiana danxiaensis* and Analysis of Potential Suitable Areas (Master’s Thesis). Central South University of Forestry and Technology.

Zhou, Y., Zhang, Z., Zhu, B., Cheng, X., Yang, L., Gao, M., Kong, R., 2021. MaxEnt Modeling Based on CMIP6 Models to Project Potential Suitable Zones for *Cunninghamia lanceolata* in China. Forests 12, 752. https://doi.org/10.3390/f12060752

# Supplementary Figures and Tables

**Supplementary Table 1 | Climate factor**

| **Climate factor** | **Description** |
| --- | --- |
| BIO01 | Annual mean temperature (°C×10) |
| BIO02 | Mean diurnal range (Mean of monthly (max temp - min temp)) (°C×10) |
| BIO03 | Isothermality (BIO02/BIO07) (×100) |
| BIO04 | Temperature seasonality (standard deviation ×100) |
| BIO05 | Max temperature of warmest month (°C×10) |
| BIO06 | Min temperature of coldest month (°C×10) |
| BIO07 | Temperature annual range (BIO05-BIO06) (°C×10) |
| BIO09 | Mean temperature of driest quarter (°C×10) |
| BIO10 | Mean temperature of warmest quarter (°C×10) |
| BIO11 | Mean temperature of coldest quarter (°C×10) |
| BIO12 | Annual precipitation (mm) |
| BIO13 | Precipitation of wettest month (mm) |
| BIO14 | Precipitation of driest month (mm) |
| BIO15 | Precipitation seasonality (Coefficient of Variation) (mm) |
| BIO16 | Precipitation of wettest quarter (mm) |
| BIO17 | Precipitation of driest quarter (mm) |
| BIO18 | Precipitation of warmest quarter (mm) |
| BIO19 | Precipitation of coldest quarter (mm) |

**Supplementary Table 2 | Floristic region**

| **Floristic region** | **Description** |
| --- | --- |
| **2** | Pantropic |
| **3** | Trop. Asia & Trop. Amer. disjuncted |
| **4** | Old World Tropics |
| **4-1** | Trop. Asia, Africa (or E. Afr., Madagascar) & Australasia disjuncted. |
| **5** | Tropical Asia & Trop. Australasia |
| **6** | Trop. Asia to Trop. Africa |
| **7** | Trop. Asia (Indo-Malesia) |
| **7-1** | Java (or Sumatra) & Himalaya to South & Southwest China disjuncted or diffused |
| **7-4** | Vietnam (or Indo Chinese Peninsula) to S. China (or SW. China) |
| **8** | North Temperate |
| **8-4** | N. Temp. & S. Temp. disjuncted |
| **9** | E. Asia & N. Amer. disjuncted |
| **10** | Old World Temperate |
| **10-1** | Mediterranea. W. Asia (or C. Asia) & E. Asia disjuncted |
| **12** | Mediterranea, W. Asia to C. Asia |
| **13-1** | East C. Asia (or Asia Media), in Sinkiang (especially Kaschgaria)， Kansu, Qinghai to Mongolia. |
| **14** | E. Asia |
| **14SH** | Sino-Himalaya |
| **15** | Endemic to China |

**Supplementary Table 3 | Effect sizes (categorical moderators and** **continuous moderators) for the overall suitable region area and the highly suitable region area.**

|  | **The overall suitable region area** | | | | |  | **The highly suitable region area** | | | | |
| --- | --- | --- | --- | --- | --- | --- | --- | --- | --- | --- | --- |
| **Fixed effects Moderators** | **Mean effect size** | **LCI** | **UCI** | **N** | **Model support based on omnibus test (QM,df)****/Pval** |  | **Mean effect size** | **LCI** | **UCI** | **N** | **Modelsupport based on omnibus test(QM,df) /Pval** |
| **Plant life form (categorical moderators)** |  |  |  | **528** | **QM =5.0396**  **P=0.0248** |  |  |  |  |  | **QM= 5.1427 P = 0.0233** |
| arbor | 0.0152 | -0.0172 | 0.0476 | 364 | P=0.3567 |  | -0.5588 | -0.8296 | -0.2879 | 284 | P<0.0001 |
| shrub | 0.0818 | 0.0336 | 0.1301 | 164 | P=0.0009 |  | -0.0222 | -0.3986 | 0.3543 | 147 | P=0.9081 |
| **Floristic region**  **(categorical moderators)** |  |  |  |  | **QM = 75.6180  P < 0.0001** |  |  |  |  |  | **QM= 36.8910 P = 0.0054** |
| 2 | 0.1034 | 0.0102 | 0.1965 | 40 | P=0.0296 |  | 0.0461 | -0.7126 | 0.8049 | 35 | P=0.9052 |
| 3 | 0.0165 | -0.109 | 0.142 | 22 | P=.7967 |  | -3.03 | -4.0337 | -2.0263 | 20 | P<0.0001 |
| 4 | 0.2547 | 0.0143 | 0.495 | 6 | P=0.0378 |  | 0.773 | -1.0596 | 2.6055 | 6 | P=0.4084 |
| 4-1 | 0.1188 | -0.1218 | 0.3595 | 6 | P=0.333 |  | -0.0211 | -1.8537 | 1.8114 | 6 | P=0.982 |
| 5 | 0.0065 | -0.141 | 0.1540 | 16 | P=0.931 |  | 0.3392 | -0.9566 | 1.6350 | 12 | P=0.6079 |
| 6 | 0.3635 | 0.0694 | 0.6577 | 4 | P=0.0154 |  | -0.4806 | -2.7250 | 1.7638 | 4 | P=0.6747 |
| 7 | 0.4583 | 0.2719 | 0.6448 | 10 | P <0.0001 |  | 0.4089 | -1.0106 | 1.8285 | 10 | P=0.5723 |
| 7-1 | -0.0979 | -0.3934 | 0.1975 | 4 | P=0.516 |  | 0.3442 | -2.2476 | 2.9360 | 3 | P=0.7946 |
| 7-4 | -0.0593 | -0.4757 | 0.3571 | 2 | P=0.7802 |  | 0.3308 | -2.8433 | 3.5049 | 2 | P=0.8381 |
| 8 | 0.0020 | -0.0403 | 0.0443 | 194 | P=0.9253 |  | -0.4743 | -0.8136 | -0.1350 | 175 | P=0.0062 |
| 8-4 | 0.1759 | 0.0504 | 0.3014 | 22 | P=0.006 |  | -0.0272 | -1.4467 | 1.3922 | 10 | P=0.97 |
| 9 | 0.0237 | -0.0597 | 0.1070 | 50 | P=0.5778 |  | -0.1452 | -0.8734 | 0.5830 | 38 | P=0.696 |
| 10 | -0.4526 | -0.6488 | -0.2563 | 9 | P <0.0001 |  | -1.3463 | -2.7658 | 0.0732 | 10 | P=0.063 |
| 10-1 | 0.0490 | -0.1284 | 0.2265 | 11 | P=0.5881 |  | -0.3147 | -1.8109 | 1.1816 | 9 | P=0.6802 |
| 12 | 0.0236 | -0.2167 | 0.2640 | 6 | P=0.8473 |  | -0.0075 | -1.8400 | 1.8251 | 6 | P=0.9936 |
| 13-1 | 0.2675 | 0.0974 | 0.4376 | 12 | P=0.0021 |  | -0.4377 | -1.7336 | 0.8581 | 12 | P=0.5079 |
| 14 | 0.1504 | 0.0074 | 0.2933 | 17 | P=0.0392 |  | 0.1078 | -1.0144 | 1.2300 | 16 | P=0.8507 |
| 14SH | -0.0828 | -0.2349 | 0.0694 | 15 | P=0.2862 |  | -0.2262 | -2.4707 | 2.0183 | 4 | P=0.8434 |
| 15 | 0.0002 | -0.0648 | 0.0652 | 82 | P=0.9946 |  | -0.1103 | -0.7269 | 0.5063 | 53 | P=0.726 |
| **Plant Genus**  **(categorical moderators)** |  |  |  |  | **QM = 357.0119,**  **P <0 .0001** |  |  |  |  |  | **QM = 189.6463,**  **P < .0001** |
| *Abies* | 0.3214 | -0.1796 | 0.8224 | 1 | P=0.2086 |  | 0.1888 | -3.828 | 4.2056 | 1 | P=0.9266 |
| *Acer* |  |  |  |  |  |  | -0.6629 | -2.9819 | 1.6562 | 3 | P=0.5753 |
| *Alsophila* | 0.0656 | -0.123 | 0.2541 | 7 | P=0.4955 |  | -0.2376 | -1.7558 | 1.2805 | 7 | P=0.759 |
| *Altingia* | -0.0979 | -0.3486 | 0.1528 | 4 | P=0.444 |  | 0.3442 | -1.9749 | 2.6634 | 3 | P=0.7711 |
| *Ammopiptanthus* | 0.3684 | 0.1918 | 0.545 | 8 | P<0.0001 |  | -0.4078 | -1.8279 | 1.0123 | 8 | P=0.5736 |
| *Artemisia* | -0.1132 | -0.3627 | 0.1363 | 4 | P=0.3738 |  | -0.2272 | -2.2355 | 1.7811 | 4 | P=0.8245 |
| *Brachychiton* | -0.4602 | -0.8127 | -0.1077 | 2 | P=0.0105 |  |  |  |  |  |  |
| *Calligonum* | 0.0236 | -0.18 | 0.2273 | 6 | P=0.8202 |  | -0.0075 | -1.6472 | 1.6323 | 6 | P=0.9929 |
| *Camellia* | 0.674 | 0.4854 | 0.8625 | 7 | P<0.0001 |  | 0.5876 | -0.9305 | 2.1058 | 7 | P=0.448 |
| *Carpinus* | -0.0855 | -0.2432 | 0.0722 | 10 | P=0.2882 |  | -0.7328 | -1.8063 | 0.3407 | 14 | P=0.1809 |
| *Cassia* | 0.5236 | 0.1713 | 0.876 | 2 | P=0.0036 |  | 0.7404 | -3.2761 | 4.7569 | 1 | P=0.7179 |
| *Catalpa* | 0.2155 | 0.0387 | 0.3923 | 8 | P=0.0169 |  | 0.0404 | -1.3797 | 1.4605 | 8 | P=0.9555 |
| *Cathaya* | -0.0031 | -0.1474 | 0.1411 | 12 | P=0.9662 |  | -0.8789 | -2.8873 | 1.1295 | 4 | P=0.391 |
| *Cephalotaxus* | 0.1504 | 0.0293 | 0.2715 | 17 | P=0.015 |  | 0.1078 | -0.8964 | 1.1119 | 16 | P=0.8334 |
| *Cercidiphyllum* | -0.0739 | -0.2501 | 0.1023 | 8 | P=0.4109 |  |  |  |  |  |  |
| *Choerospondias* | 0.2314 | -0.2672 | 0.7299 | 1 | P=0.3631 |  |  |  |  |  |  |
| *Corylus* | 0.0429 | -0.1149 | 0.2006 | 10 | P=0.5942 |  | 0.3208 | -0.9494 | 1.5909 | 10 | P=0.6206 |
| *Cunninghamia* | -0.033 | -0.1504 | 0.0845 | 18 | P=0.5823 |  | 0.0115 | -0.9352 | 0.9582 | 18 | P=0.981 |
| *Cupressus* | 0.4406 | 0.0878 | 0.7935 | 2 | P=0.0144 |  | 1.1431 | -1.697 | 3.9833 | 2 | P=0.4302 |
| *Dalbergia* | 0.6892 | 0.4994 | 0.8791 | 7 | P<0.0001 |  | 0.5888 | -1.2076 | 2.3852 | 5 | P=0.5206 |
| *Davidia* | -0.0787 | -0.2075 | 0.05 | 15 | P=0.2308 |  | 0.087 | -1.4311 | 1.6052 | 7 | P=0.9105 |
| *Delonix* | 0.3248 | -0.0275 | 0.6771 | 2 | P=0.0708 |  | -1.0397 | -3.8798 | 1.8004 | 2 | P=0.4731 |
| *Disanthus* | -0.2393 | -0.5944 | 0.1158 | 2 | P=0.1866 |  |  |  |  |  |  |
| *Erythrophleum* | 0.1188 | -0.0851 | 0.3228 | 6 | P=0.2534 |  | -0.0211 | -1.6609 | 1.6186 | 6 | P=0.9798 |
| *Firmiana* | -0.044 | -0.3329 | 0.245 | 3 | P=0.7655 |  | -0.1871 | -2.5062 | 2.132 | 3 | P=0.8743 |
| *Fokienia* | 0.1054 | -0.3935 | 0.6044 | 1 | P=0.6787 |  | 0.8704 | -3.1462 | 4.887 | 1 | P=0.671 |
| *Forsythia* | 0.0599 | -0.1063 | 0.2262 | 9 | P=0.4797 |  | -0.3147 | -1.6535 | 1.0242 | 9 | P=0.6451 |
| *Garuga* | -0.1935 | -0.4442 | 0.0572 | 4 | P=0.1303 |  |  |  |  |  |  |
| *Ginkgo* | -0.0178 | -0.194 | 0.1584 | 8 | P=0.843 |  |  |  |  |  |  |
| *Hippophae* | -0.4526 | -0.6189 | -0.2863 | 9 | P<0.0001 |  | -1.3463 | -2.6164 | -0.0762 | 10 | P=0.0378 |
| *Hydrangea* | 0.1166 | -0.0869 | 0.3201 | 6 | P=0.2615 |  | 0.2866 | -1.3531 | 1.9264 | 6 | P=0.7319 |
| *Jacaranda* | -0.1697 | -0.5221 | 0.1826 | 2 | P=0.345 |  |  |  |  |  |  |
| *Keteleeria* | -0.2744 | -0.7739 | 0.2252 | 1 | P=0.2817 |  | -0.3434 | -4.36 | 3.6733 | 1 | P=0.8669 |
| *Larix* | -0.0053 | -0.1386 | 0.128 | 14 | P=0.9378 |  | 0.319 | -1.1011 | 1.739 | 8 | P=0.6598 |
| *Liriodendron* | 0.0357 | -0.0976 | 0.1689 | 14 | P=0.5999 |  | 0.0669 | -1.3532 | 1.4869 | 8 | P=0.9265 |
| *Lithocarpus* | -0.0467 | -0.2048 | 0.1114 | 10 | P=0.5629 |  | -0.0288 | -1.299 | 1.2414 | 10 | P=0.9646 |
| *Litsea* | 0.0376 | -0.1065 | 0.1816 | 12 | P=0.6092 |  | -1.8111 | -2.9706 | -0.6516 | 12 | P=0.0022 |
| *Lonicera* | -0.1102 | -0.249 | 0.0286 | 13 | P=0.1197 |  | -0.2279 | -1.3419 | 0.8862 | 13 | P=0.6885 |
| *Lycium* | 0.1676 | 0.0501 | 0.2852 | 18 | P=0.0052 |  | -0.0272 | -1.2974 | 1.2429 | 10 | P=0.9665 |
| *Metasequoia* | 0.3198 | 0.0702 | 0.5693 | 4 | P=0.012 |  | 0.3879 | -1.6205 | 2.3962 | 4 | P=0.705 |
| *Myrica* | 0.2134 | -0.0359 | 0.4626 | 4 | P=0.0934 |  |  |  |  |  |  |
| *Nothotsuga* | -0.438 | -0.6876 | -0.1884 | 4 | P=0.0006 |  | -1.9326 | -3.9409 | 0.0757 | 4 | P=0.0593 |
| *Ormosia* | -0.1524 | -0.3101 | 0.0052 | 10 | P=0.0581 |  | -0.206 | -1.4762 | 1.0641 | 10 | P=0.7506 |
| *Osmanthus* | 0.049 | -0.2012 | 0.2992 | 4 | P=0.7012 |  | -0.0378 | -2.878 | 2.8025 | 2 | P=0.9792 |
| *Ostrya* | -0.3687 | -0.6593 | -0.0782 | 3 | P=0.0129 |  | -2.1728 | -4.1813 | -0.1642 | 4 | P=0.034 |
| *Ostryopsis* | -0.0544 | -0.2579 | 0.1492 | 6 | P=0.6007 |  | -0.1835 | -1.8233 | 1.4562 | 6 | P=0.8264 |
| *Paeonia* | 0.0198 | -0.2301 | 0.2698 | 4 | P=0.8763 |  | 0.3726 | -1.6357 | 2.381 | 4 | P=0.7161 |
| *Parashorea* | -0.0497 | -0.3395 | 0.2402 | 3 | P=0.7369 |  | -0.0081 | -2.3273 | 2.3111 | 3 | P=0.9945 |
| *Phoebe* | 0.0315 | -0.1448 | 0.2078 | 8 | P=0.726 |  | -4.8583 | -6.2784 | -3.4383 | 8 | P<0.0001 |
| *Picea* | 0.1641 | 0.0601 | 0.2681 | 23 | P=0.002 |  | -0.1409 | -0.9784 | 0.6966 | 23 | P=0.7416 |
| *Pinus* | -0.0571 | -0.161 | 0.0469 | 23 | P=0.2818 |  | 0.1359 | -0.684 | 0.9558 | 24 | P=0.7453 |
| *Populus* | -0.7011 | -0.9242 | -0.478 | 5 | P<0.0001 |  | -0.5894 | -2.3857 | 1.2068 | 5 | P=0.5201 |
| *Potaninia* | 0.0659 | -0.1838 | 0.3155 | 4 | P=0.6051 |  | -0.4976 | -2.5059 | 1.5107 | 4 | P=0.6272 |
| *Prunus* | -0.0491 | -0.2068 | 0.1085 | 10 | P=0.5414 |  | 0.1446 | -1.1256 | 1.4147 | 10 | P=0.8235 |
| *Quercus* | 0.0147 | -0.1292 | 0.1586 | 12 | P=0.841 |  | -0.0288 | -1.1883 | 1.1307 | 12 | P=0.9612 |
| *Rhododendron* | 0.0283 | -0.0786 | 0.1353 | 22 | P=0.6036 |  | 0.3917 | -1.248 | 2.0315 | 6 | P=0.6396 |
| *Rhodomyrtus* | 0.2318 | 0.0283 | 0.4353 | 6 | P=0.0256 |  |  |  |  |  |  |
| *Rosa* | -0.0348 | -0.1927 | 0.1232 | 10 | P=0.666 |  | -0.4572 | -1.7273 | 0.813 | 10 | P=0.4805 |
| *Salix* | 0.5498 | 0.346 | 0.7536 | 6 | P<.0001 |  | 0.4588 | -1.181 | 2.0985 | 6 | P=0.5834 |
| *Sassafras* | 0.0709 | -0.1784 | 0.3202 | 4 | P=0.5772 |  |  |  |  |  |  |
| *Semiliquidambar* | -0.2264 | -0.4758 | 0.023 | 4 | P=0.0752 |  |  |  |  |  |  |
| *Sorbus* | -0.1709 | -0.329 | -0.0127 | 10 | P=0.0342 |  | -0.048 | -1.3182 | 1.2222 | 10 | P=0.941 |
| *Spathodea* | 0.4023 | 0.0499 | 0.7546 | 2 | P=0.0252 |  | 0.0785 | -2.7616 | 2.9186 | 2 | P=0.9568 |
| *Taiwania* | -0.1311 | -0.3197 | 0.0574 | 7 | P=0.1729 |  | -1.5989 | -3.2387 | 0.0408 | 6 | P=0.056 |
| *Taxus* | 0.1306 | -0.1188 | 0.38 | 4 | P=0.3047 |  | 0.0849 | -1.9234 | 2.0932 | 4 | P=0.934 |
| *Tetradium* | 0.2547 | 0.051 | 0.4583 | 6 | P=0.0142 |  | 0.773 | -0.8668 | 2.4127 | 6 | P=0.3555 |
| *Toona* | -0.0871 | -0.2919 | 0.1177 | 6 | P=0.4045 |  | 0.2867 | -1.3532 | 1.9266 | 6 | P=0.7319 |
| *Tsoongiodendron* | -0.224 | -0.723 | 0.2749 | 1 | P=0.3789 |  | -0.2088 | -4.2254 | 3.8078 | 1 | P=0.9189 |
| *Ulmus* | 0.1975 | 0.021 | 0.374 | 8 | P=0.0283 |  | -8.0164 | -9.4365 | -6.5964 | 8 | P<0.0001 |
| *Xanthoceras* | 0.3554 | 0.1791 | 0.5318 | 8 | P<0.0001 |  | 0.7498 | -0.6702 | 2.1699 | 8 | P=0.3007 |
| *Zelkova* | 0 | -0.3525 | 0.3525 | 2 | P=1 |  |  |  |  |  |  |
| *Ziziphus* | 0.0235 | -0.1204 | 0.1673 | 12 | P=0.7492 |  | 0.1378 | -1.0217 | 1.2973 | 12 | P=0.8158 |
| **Greenhouse gas concentration path**  **(categorical moderators)** |  |  |  |  | **QM= 14.2850, P = 0.0463** |  |  |  |  |  | **QM= 16.6432 P = 0.0198** |
| RCP2.6 | 0.0049 | -0.0517 | 0.0614 | 119 | P=0.8662 |  | -0.3718 | -0.8525 | 0.109 | 89 | P=0.1297 |
| RCP4.5 | 0.043 | -0.0152 | 0.1013 | 112 | P=0.1478 |  | -0.4873 | -0.9708 | -0.0037 | 88 | P=0.0483 |
| RCP6.0 | 0.0016 | -0.1128 | 0.116 | 29 | P=0.9778 |  | -2.3298 | -3.3704 | -1.2892 | 19 | P=<0.0001 |
| RCP8.5 | 0.0034 | -0.0492 | 0.0561 | 137 | P=0.8984 |  | -0.3289 | -0.7759 | 0.118 | 103 | P=0.1492 |
| SSP126 | 0.0302 | -0.0673 | 0.1277 | 40 | P=0.5438 |  | -0.0632 | -0.7716 | 0.6451 | 41 | P=0.8611 |
| SSP245 | 0.2027 | 0.0984 | 0.3069 | 35 | P=0.0001 |  | 0.0899 | -0.6880 | 0.8678 | 34 | P=0.8208 |
| SSP370 | 0.1019 | -0.0572 | 0.2609 | 15 | P=0.2095 |  | 0.0609 | -1.1102 | 1.2321 | 15 | P=0.9188 |
| SSP585 | 0.0788 | -0.0174 | 0.1751 | 41 | P=0.1084 |  | -0.2194 | -0.9193 | 0.4805 | 42 | P=0.5389 |
| **Climate factor**  **(continuous moderators)** |  |  |  |  |  |  |  |  |  |  |  |
| **BIO01** | 0.4108 | 0.1957 | 0.6260 | 69 | QM= 14.0064, P = 0.0002 |  | 0.8380 | -1.0048 | 2.6808 | 54 | QM= 0.7945, P = 0.3728 |
| **BIO02** | -0.4695 | -0.7226 | -0.2165 | 107 | QM= 13.2270 P = 0.0003 |  | 0.1104 | -2.3829 | 2.6037 | 64 | QM= 0.0075, P = 0.9308 |
| **BIO03** | 0.8423 | 0.3575 | 1.3271 | 34 | QM= 11.5966 P = 0.0007 |  | 1.1722 | -3.1874 | 5.5317 | 31 | QM= 0.2777, P = 0.5982 |
| **BIO04** | 0.0175 | -0.1526 | 0.1876 | 133 | QM= 0.0407 P = 0.8401 |  | -4.6129 | -6.0182 | -3.2075 | 99 | QM= 41.3876, P < .0001 |
| **BIO05** | 0.2646 | -0.1019 | 0.6311 | 20 | QM= 2.0021 P = 0.1571 |  | 2.0081 | -0.6909 | 4.7071 | 21 | QM= 2.1265, P = 0.1448 |
| **BIO06** | 0.0465 | -0.0662 | 0.1592 | 108 | QM= 0.6532 P = 0.4190 |  | -0.1745 | -1.0287 | 0.6797 | 95 | QM= 0.1603, P = 0.6889 |
| **BIO07** | -0.0827 | -0.2895 | 0.1241 | 60 | QM= 0.6144 P = 0.4331 |  | 1.3532 | -0.9860 | 3.6924 | 34 | QM= 1.2855, P = 0.2569 |
| **BIO09** | 0.1737 | -0.1353 | 0.4827 | 59 | QM= 1.2139 P = 0.2706 |  | 1.8669 | -0.9732 | 4.7070 | 37 | QM= 1.6599, P = 0.1976 |
| **BIO10** | 0.5913 | 0.2040 | 0.9785 | 35 | QM= 8.9550 P = 0.0028 |  | 2.0925 | -0.8538 | 5.0388 | 27 | QM= 1.9377, P = 0.1639 |
| **BIO11** | 0.1972 | 0.0656 | 0.3287 | 70 | QM= 8.6326 P = 0.0033 |  | 1.1405 | 0.0930 | 2.1881 | 61 | QM = 4.5534, P = 0.0329 |
| **BIO12** | -0.0011 | -0.1168 | 0.1145 | 182 | QM= 0.0004 P = 0.9849 |  | 0.6100 | -0.3053 | 1.5252 | 144 | QM= 1.7061, P = 0.1915 |
| **BIO13** | -0.2368 | -0.4733 | -0.0004 | 51 | QM= 3.8532 P = 0.0497 |  | -0.2513 | -2.1518 | 1.6491 | 38 | QM= 0.0672, P = 0.7955 |
| **BIO14** | -0.0675 | -0.1646 | 0.0296 | 126 | QM= 1.8554 P = 0.1732 |  | 0.2686 | -0.4970 | 1.0343 | 109 | QM= 0.4729, P = 0.4917 |
| **BIO15** | -0.0728 | -0.0728 | 0.1818 | 70 | QM= 0.3141 P = 0.5752 |  | -3.0751 | -5.0900 | -1.0601 | 65 | QM= 8.9470, P = 0.0028 |
| **BIO16** | -0.2854 | -0.7519 | 0.1811 | 33 | QM= 1.4381 P = 0.2305 |  | 0.8502 | -2.6288 | 4.3291 | 33 | QM= 0.2294, P = 0.6320 |
| **BIO17** | -0.0626 | -0.2426 | 0.1174 | 56 | QM= 0.4647 P = 0.4954 |  | -0.5424 | -2.0227 | 0.9378 | 44 | QM= 0.5158, P = 0.4726 |
| **BIO18** | 0.1992 | 0.0223 | 0.3761 | 70 | QM= 4.8712 P = 0.0273 |  | 0.7849 | -0.6062 | 2.1760 | 66 | QM= 1.2230, P = 0.2688 |
| **BIO19** | 0.0509 | -0.2445 | 0.3464 | 48 | QM= 0.1141  P = 0.7356 |  | 0.5465 | -1.6518 | 2.7448 | 48 | QM = 0.2374, P = 0.6261 |

## Supplementary Figure


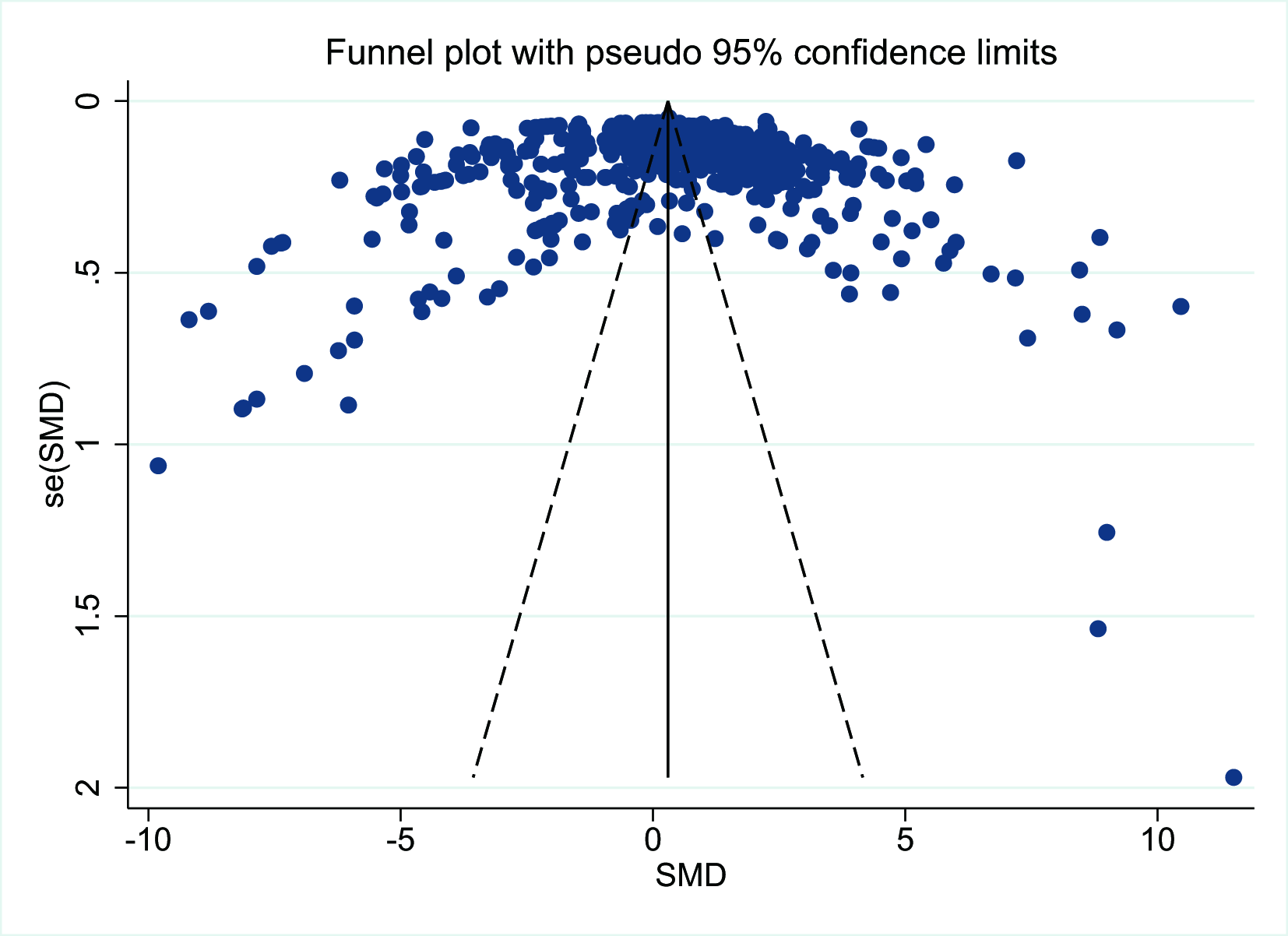


**Supplementary Figure 1 |** Funnel plot of the highly suitable area.


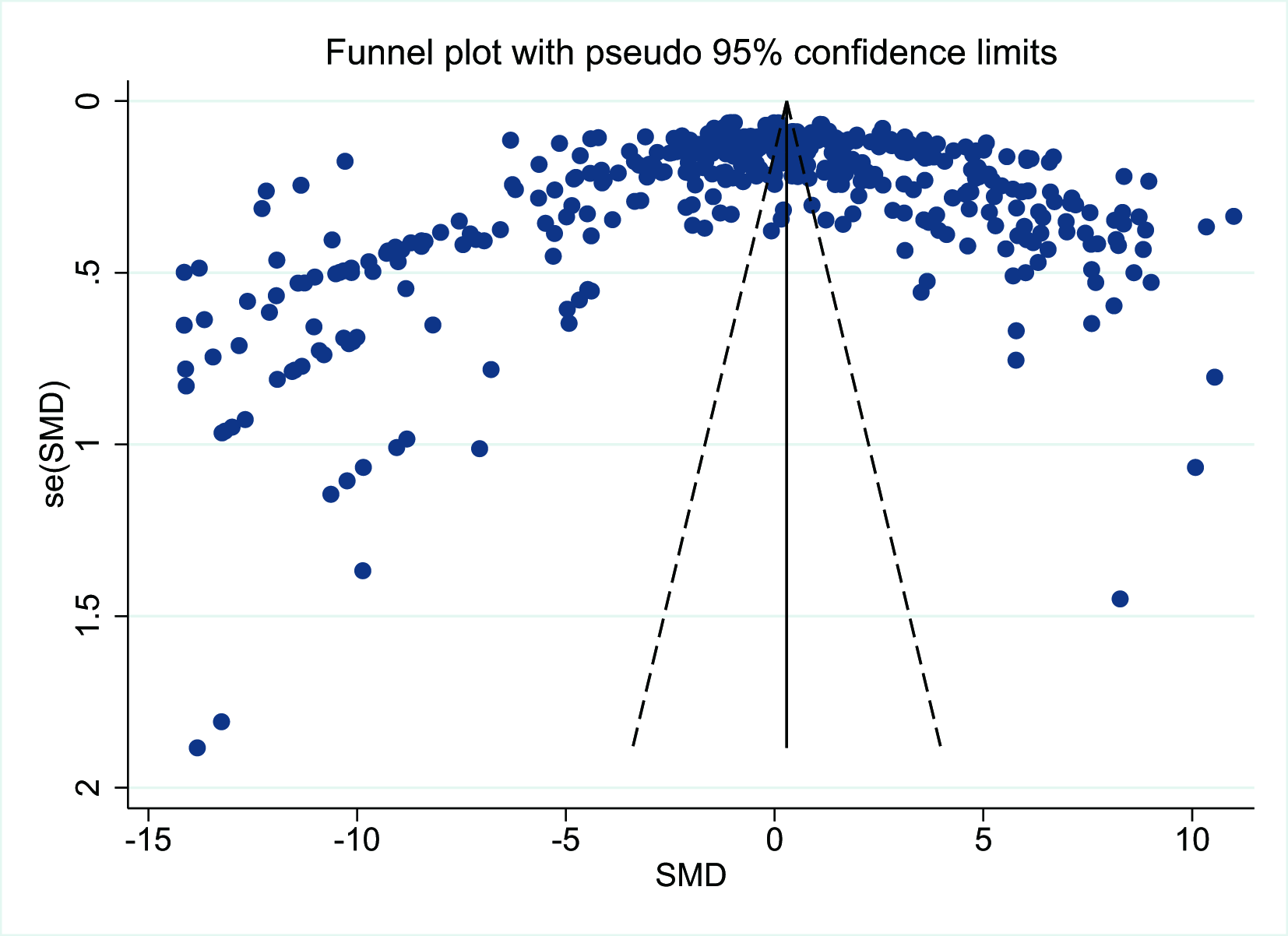


**Supplementary Figure 2 |** Funnel plot of the highly suitable area
